# European stroke organisation and European society for minimally invasive neurological therapy guideline on acute management of basilar artery occlusion

**DOI:** 10.1177/23969873241257223

**Published:** 2024-07-22

**Authors:** Daniel Strbian, Georgios Tsivgoulis, Johanna Ospel, Silja Räty, Petra Cimflova, Georgios Georgiopoulos, Teresa Ullberg, Caroline Arquizan, Jan Gralla, Kamil Zeleňák, Salman Hussain, Jens Fiehler, Patrik Michel, Guillaume Turc, Wim Van Zwam

**Affiliations:** 1 Department of Neurology, Helsinki University Central Hospital, Helsinki, Finland; 2 Second Department of Neurology, ‘Attikon’ University Hospital of Athens, National and Kapodistrian University of Athens, Athens, Greece; 3 Neuroradiology, Department of Diagnostic Imaging, Foothills Medical Center, University of Calgary, Calgary, AB, Canada; 4 Foothills Medical Center, University of Calgary, Calgary, AB, Canada; 5 Department of Physiology, School of Medicine, University of Patras, Greece; 6 School of Biomedical Engineering and Imaging Sciences, King’s College London, London, UK; 7 Department of Clinical Sciences Lund, Lund University, Skane University Hospital, Lund and Malmö, Malmö, Sweden; 8 Department of Neurology, Hôpital Gui de Chauliac, INSERM U1266, Montpellier, France; 9 Neuroradiology, Inselspital, University of Bern, Bern, Switzerland; 10 Clinic of Radiology, Jessenius Faculty of Medicine, Comenius University, Martin, Slovakia; 11 European Stroke Organisation, Basel, Switzerland; 12 UMC Hamburg-Eppendorf, Hamburg, Germany; 13 Department of Clinical Neuroscience, Lausanne University Hospital and University of Lausanne, Bâtiment Hospitalier Principal, Lausanne, Switzerland; 14 Department of Neurology, GHU Paris Psychiatrie et Neurosciences, INSERM U1266, Université Paris Cité, FHU NeuroVasc, Paris, France; 15 Department of Radiology and Nuclear Medicine, Maastricht University Medical Centre, Maastricht, The Netherlands

**Keywords:** Guideline, systematic review, stroke, basilar artery occlusion, posterior circulation, acute management, thrombolysis, endovascular treatment

## Abstract

The aim of the present European Stroke Organisation (ESO) guideline is to provide evidence-based recommendations on the acute management of patients with basilar artery occlusion (BAO). These guidelines were prepared following the Standard Operational Procedure of the ESO and according to the GRADE methodology. Although BAO accounts for only 1%–2% of all strokes, it has very poor natural outcome. We identified 10 relevant clinical situations and formulated the corresponding Population Intervention Comparator Outcomes (PICO) questions, based on which a systematic literature search and review was performed. The working group consisted of 10 voting members (five representing ESO and five ESMINT) and three non-voting junior members. The certainty of evidence was generally very low. In many PICOs, available data were scarce or lacking, hence, we provided expert consensus statements. First, we compared intravenous thrombolysis (IVT) to no IVT, but specific BAO-related data do not exist. Yet, historically, IVT was standard of care for BAO patients who were also included (albeit in small numbers) in IVT trials. Non-randomised studies of IVT-only cohorts showed high proportion of favourable outcomes. Expert Consensus suggests using IVT up to 24 h unless otherwise contraindicated. We further suggest IVT plus endovascular treatment (EVT) over direct EVT. EVT on top of best medical treatment (BMT) was compared to BMT alone within 6 and 6–24 h from last seen well. In both time windows, we observed a different effect of treatment depending on (a) the region where the patients were treated (Europe vs. Asia), (b) on the proportion of IVT in the BMT arm, and (c) on the initial stroke severity. In case of high proportion of IVT in the BMT group and in patients with NIHSS below 10, EVT plus BMT was not found better than BMT alone. Based on very low certainty of evidence, we suggest EVT + BMT over BMT alone (i.e. based on results of patients with at least 10 NIHSS points and a low proportion of IVT in BMT). For patients with an NIHSS below 10, we found no evidence to recommend EVT over BMT. In fact, BMT was non-significantly better and safer than EVT. Furthermore, we found a stronger treatment effect of EVT + BMT over BMT alone in proximal and middle locations of BAO compared to distal location. While recommendations for patients without extensive early ischaemic changes in the posterior fossa can, in general, follow those of other PICOs, we formulated an Expert Consensus Statement suggesting against reperfusion therapy in those with extensive bilateral and/or brainstem ischaemic changes. Another Expert Consensus suggests reperfusion therapy regardless of collateral scores. Based on limited evidence, we suggest direct aspiration over stent retriever as the first-line strategy of mechanical thrombectomy. As an Expert Consensus, we suggest rescue percutaneous transluminal angioplasty and/or stenting after a failed EVT procedure. Finally, based on very low certainty of evidence, we suggest add-on antithrombotic treatment during EVT or within 24 h after EVT in patients with no concomitant IVT and in whom EVT was complicated (defined as failed or imminent re-occlusion, or need for additional stenting or angioplasty).

## Introduction

Basilar artery occlusion (BAO) comprises only 1%–2% of ischaemic stroke but imposes a significant burden on patients due to the associated high disability and mortality.^1,2^ Reperfusion therapy is the standard of care for improving outcome of eligible patients with acute ischaemic stroke. The European Stroke Organisation (ESO) Guideline on intravenous thrombolysis (IVT) does not differentiate recommendations based on stroke location.^3^ Accordingly, IVT is an integral part of acute management of BAO despite the lack of randomised controlled trials (RCT) focusing specifically on posterior circulation occlusions. Very poor prognosis of untreated BAO is probably the most important reason for not having pivotal RCTs comparing IVT to no reperfusion therapy. Evidence for the efficacy of EVT has until recently been mainly confined to anterior circulation large-vessel occlusions.^4^ Consequently, the 2019 joint Guideline of the ESO and the European Society for Minimally Invasive Neurological Therapy (ESMINT) on mechanical thrombectomy in AIS could only constitute an expert opinion on EVT in BAO,^5^ leaving considerable uncertainty about the optimal acute management of the disease.

Since 2019, four RCTs on EVT plus best medical treatment (BMT) versus BMT for acute BAO have been published.^6–9^ This has generated the need to systematically compile the current evidence from RCTs and observational studies on reperfusion therapy exclusively for BAO. The aim of this ESO-ESMINT Guideline is to provide evidence-based recommendations to assist stroke physicians in their decision-making in the acute management of BAO. However, the number of available RCTs is rather small and geographical differences are considerable. For example, the high prevalence of intracranial atherosclerotic disease (ICAD) in Asian population, and a significantly higher proportion of IVT in BMT in the European trial. For these reasons, we also included data from nonrandomised studies of interventions (NRSIs).

In general, there are five relevant justifications for including NRSIs in a systematic review along with RCTs.^10,11^ The two main reasons are (1) the evidence can be studied in RCTs, but the trials address the review question indirectly or incompletely (in these cases, NRSIs might better match the review question), and (2) interventions that *cannot* be randomised, or that are extremely unlikely to be studied in RCTs. Both of these reasons apply to our guidelines, where three of the four RCTs were performed in Asian population, and the outcome of their BMT arm differed significantly from the BMT arm of the European RCT. Proportion of IVT in the Asian trials was very low compared to the European trial, and it is very likely that a new target RCT is neither feasible nor ethical in the near future.

All precautions were taken to properly assess the risk of bias both in the RCTs (RoB 2, Cochrane^11^) and the NRSI (ROBINS-I^10^). Furthermore, every effort was made to evaluate (a) whether NRSI has the study design features required to address a particular Population Intervention Comparator Outcomes (PICO) question and (b) whether it directly addresses the PICO question (regarding intervention, comparator, outcome, and setting).

## Methods

### Composition and approval of the Module Working Group

These guidelines were initiated by the ESO and drawn up in cooperation with the ESMINT. Daniel Strbian and Wim van Zwam were selected as chairpersons to assemble and coordinate the Guideline Module Working Group (MWG). The final group contained five stroke neurologists from the ESO and five interventional radiologists from the ESMINT. In addition, three non-voting fellows were selected both from the ESO and the ESMINT. Of all MWG members, five were females. The ESO Guideline Board and the Executive Committees of the ESO and the ESMINT reviewed the intellectual and financial disclosures of all MWG members and approved the composition of the group. Full details of all MWG members and their disclosures are included in the Supplemental Table 1.

### Development and approval of clinical questions

This guideline was prepared according to the ESO standard operating procedures (SOP),^12^ which are based on the Grading of Recommendations, Assessment, Development and Evaluations (GRADE) framework. The MWG developed a list of topics and corresponding questions of greatest clinical interest. Questions were formatted using the PICO approach and reviewed by two external reviewers as well as members of the ESO Guideline board and Executive Committee. The outcomes were rated by the members of the MWG as critical, important, or of limited importance according to the GRADE criteria. The final decision on outcomes used a Delphi approach. The results of the outcome rating for each PICO question are included in the Supplemental Table 2.

Based on the recent STAIR guidance,^13^ the following wording was used to describe the modified Rankin Scale (mRS) score outcomes: mRS 0–1: excellent outcome; mRS 0–2: good outcome; mRS 0–3: moderate outcome; shift/ordinal analysis of the mRS: reduced disability (reduction of at least 1 point over the mRS at 90 days).

## Literature search

For each PICO question, search terms were prepared by the MWG and a guideline methodologist. Where an existing and validated search strategy was available (e.g. from an existing systematic review), it was used or adapted. If a question of interest had recently undergone an appropriate systematic review, the corresponding search strategy and identified references were used, combined, and updated as necessary. The search strategies are described in Supplemental Table 3.

The search per se was conducted by the ESO Guideline methodologist Salman Hussain. The Ovid Medline and Embase databases were searched from the inception to 13 January 2023. Reference lists of review articles, authors’ personal reference libraries, and previous guidelines were also searched for additional relevant records. The search was validated with multiple references provided for the validation process by all MWG members and matched each specific PICO question. Finally, the search was updated in PubMed until 20 February 2024.

The search results from Medline and Embase were uploaded to the web-based Covidence platform (Health Innovation, Melbourne, Australia) for review by the MWG. Two or more MWG members were assigned to independently screen the titles and abstracts of publications registered in the Covidence platform and then evaluate the full text of potentially relevant studies. Any disagreements were resolved by discussion between two reviewers or a third MWG member (including one of the chairpersons).

RCTs were prioritised, but due to limited randomised data, health registry data analyses, observational studies (minimum size: 20 subjects), and systematic reviews or meta-analyses of observational studies were also considered. Only angiography-verified BAO studies in adults published in English were considered. We excluded publications of only abstracts and protocols.

### Data analysis

Data extraction was performed by all members of the MWG and data analysis was performed by Georgios Georgiopoulos, Daniel Strbian, and Georgios Tsivgoulis. If relevant data were not reported in an eligible study, the corresponding author was contacted. In case of no response, the co-authors of the study were also contacted and reminded twice. If no answer was received, the data were considered missing.

Cochrane and GRADE recommendations for meta-analyses were followed, including both RCT and NRSI studies.^14^ Random-effects meta-analyses were conducted using Review Manager (RevMan) software (Cochrane). In rare cases, the rate ratio was reported in the original paper of some studies, and it was considered an approximation of the risk ratio (RR) (we used a footnote of the figure to report such a step). Results were presented as estimates of effect with associated 95% confidence intervals (95% CIs). Statistical heterogeneity across studies beyond random error was quantified using the *I*^2^ statistic, and classified as:

- 0%–40%: might not be important
- 30%–60%: may represent moderate heterogeneity
- 50%–90%: may represent substantial heterogeneity
- 75%–100%: considerable heterogeneity.

The importance of the observed value of *I*^2^ depends on (1) the magnitude and direction of effects, and (2) the strength of evidence for heterogeneity (e.g. *p*-value from the Chi^2^ test, or a confidence interval for *I*^2^: uncertainty in the value of *I*^2^ is substantial when the number of studies is small).^15^

For some PICOs, prespecified subgroup analyses of ethnicity, composition of the BMT group (IVT proportion and timing of IVT administration), severity of stroke, and occlusion location were performed. We used the generic inverse-variance method in the meta-analysis. In addition, due to the expected heterogeneity among NRSIs, a random-effects meta-analysis (instead of a fixed-effect approach) was used in these guidelines as suggested as the default option.

### Evaluation of the quality of evidence and formulation of recommendations

The risk of bias of each included RCT was assessed with the Cochrane Rob2 tool.^11^ As recommended, the evidence synthesis did not use a quality ‘score’ threshold but classified overall risk of bias at study level and then in aggregate. The risk of bias of included NRSIs were assessed with the Cochrane ROBINS-I tool.^10^

The results of the data analysis were imported into the GRADEpro Guideline Development Tool (McMaster University, 2015; developed by Evidence Prime, Inc.). For each PICO question and the primary outcome, the following were considered: risk of bias based on available evidence (randomised or observational studies); considerations on inconsistency of results; indirectness of evidence, imprecision of results, and other possible bias. The GRADE evidence profiles/summary of findings tables were generated and used to prepare recommendations. ‘Evidence-based Recommendations’ were based on the GRADE methodology. The direction, strength and formulation of the recommendations were determined according to the GRADE evidence profiles and the ESO-SOP.^12,16^

Finally, expert consensus statements were added whenever the MWG members considered that there was insufficient evidence available to provide evidence-based recommendations and where practical guidance is needed for routine clinical practice. The expert consensus statements were based on voting by 10 senior expert MWG members with voting rights. Importantly, these expert consensus statements should not be regarded as evidence-based recommendations, since they only reflect the opinion of the writing group.

### Drafting of the document, revision and approval

Each PICO question is addressed in distinct sections in line with the updated ESO SOP.^12^

First, ‘Analysis of current evidence’ summarises current pathophysiological considerations followed by a summary and discussion of the results of the identified RCTs and other studies.

Second, ‘Additional information’ was provided when more details on the studies referred to in the first section has been needed to provide information on key subgroup analyses of the included studies, on ongoing or future RCTs, and on other studies, which can provide important clinical guidance on the topic.

Third, an ‘Expert Consensus Statement’ paragraph has been added whenever the MWG considered that there is insufficient evidence to make evidence-based recommendations for situations in which practical guidance is needed for everyday clinical practice.

The Guideline document was reviewed several times by all MWG members and modified using a Delphi approach until a consensus was reached. The final submitted document was peer-reviewed by two external reviewers, two members of the ESO Guideline Board and one member of the ESO Executive Committee.

## Results

### PICO 1

For adults with BAO-related acute ischaemic stroke presenting within 24 h from time last known well, does intravenous thrombolysis (IVT) alone compared to no IVT improve outcomes?

### Analysis of current evidence

The literature search did not identify any RCTs specifically addressing this PICO question, which focused on the comparison between IVT and no IVT. Although BAO was not an exclusion criterion in the pivotal IVT trials,^17–19^ it is very likely that the number of patients with BAO included in these trials was very small. This is primarily because the majority of patients enrolled in these trials did not undergo vascular imaging. Additionally, BAO accounts for only approximately 1%–2% of all AISs and is often associated with a very severe neurological deficit, which was an exclusion criterion in the ECASS trials.^19,20^ Therefore, the results of the available IVT trials cannot be directly applied to patients with acute BAO.

**Figure 1.1.**
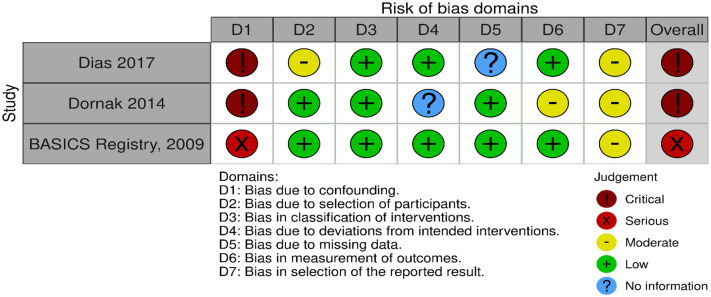
PICO 1 – Bias evaluation for the observational studies.

Our literature search identified three observational studies (all with critical bias, as shown in Figure 1.1) comparing IVT versus no IVT. These studies were included in a meta-analysis. The Basilar Artery International Cooperation Study (BASICS) international prospective registry recruited 592 consecutive patients with acute symptomatic BAO (mean age: 63, median NIHSS score: 22) between 2002 and 2007.^2^ The treatment, which was left to the discretion of each investigator, was heterogeneous and divided into three groups for the main analysis: ‘antithrombotic therapy only’ (antiplatelets or anticoagulation mostly by heparin; *n* = 183), ‘primary IVT’ (*n* = 121), which included subsequent intra-arterial thrombolysis in 41 (33.9%) patients, and ‘intra-arterial therapy only’ (*n* = 179). Functional outcome was assessed at 1 month and the presentation of the results was stratified by clinical severity (severe deficit: coma, locked-in state, tetraplegia; mild-to moderate severity: any other situation). Compared with ‘antithrombotic therapy only’, patients in the ‘primary IVT’ group tended to have a lower probability of mRS ⩾ 4 at 1 month in case of severe deficit (adjusted RR 0.88, 95% CI: 0.76–1.01) but not in case of mild-to-moderate deficit (adjusted RR: 0.94, 95% CI: 0.60–1.45; *p* for interaction not provided).

The other two identified studies were small, retrospective, and focused on outcome prediction rather than comparison of treatments, which were heterogeneous and left at the discretion of each physician.^21,22^ In each study, only a minority of patients did not receive endovascular therapy.

All three studies were deemed to have serious-to-critical level of bias (Figure 1.1), including selection bias (possibly including contraindication to IVT as a reason why IVT was not administered in the control group) and a major risk of confounding (notably confounding by indication).

No formal meta-analysis was conducted due to not only serious but critical limitations of the available studies. The MWG concludes that there is insufficient evidence to provide evidence-based recommendations on this PICO question.


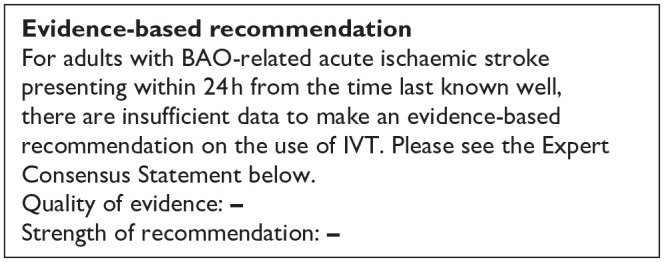


### Additional information

In this situation, where the bias of the three included observational studies is mostly critical (Figure 1.1), and results of available RCTs comparing IVT with alteplase to placebo do not directly apply to patients with acute BAO, it must be pointed out that the catastrophic prognosis of untreated BAO was the most important reason for the lack of randomised data for IVT. Consequently, many centres have considered IVT as the standard treatment for this condition for over 2 decades^2,23,24^ and it has been considered unethical to randomise patients to a trial comparing IVT with no IVT. In fact, single-arm observational data of consecutive angiography-verified BAO patients (median admission NIHSS 17) showed that up to 50% of patients achieved mRS scores of 0–3 at 3 months regardless of the time window (up to 48 h) if they presented negligible early ischaemic changes in the posterior circulation on non-contrast CT imaging (posterior circulation Alberta Stroke Program Early CT Score; pc-ASPECTS ⩾ 8).^24^ Another analysis of 245 patients (median NIHSS 18) treated with IVT alone (50% < 6 h, 19% 6–12 h, and 31% > 12 h from last-seen well) reported favourable outcome (mRS 0–3) in 47%,^25^ which is identical to the EVT arms of recent RCTs. Symptomatic intracranial haemorrhage (sICH) in that study ranged from 7% to 11%, which is in line with the data from the only RCT that used the same sICH criteria.^9^

In the BASICS registry,^2^ mRS scores of 0–2 were more frequent in the IVT group compared to the group receiving conventional treatment, with an unadjusted OR 1.83 (95% CI: 1.10–3.06). The recent ESO guidelines on IVT for AIS recommend IVT with alteplase even in AIS patients with clinically severe symptoms (NIHSS-score ⩾ 25) lasting <4.5 h (strong recommendation, moderate quality of evidence).^3^ This recommendation highlights that IVT should not be withheld from AIS patients with severe symptoms. Finally, PICO 7 addressed the role of IVT prior to EVT.


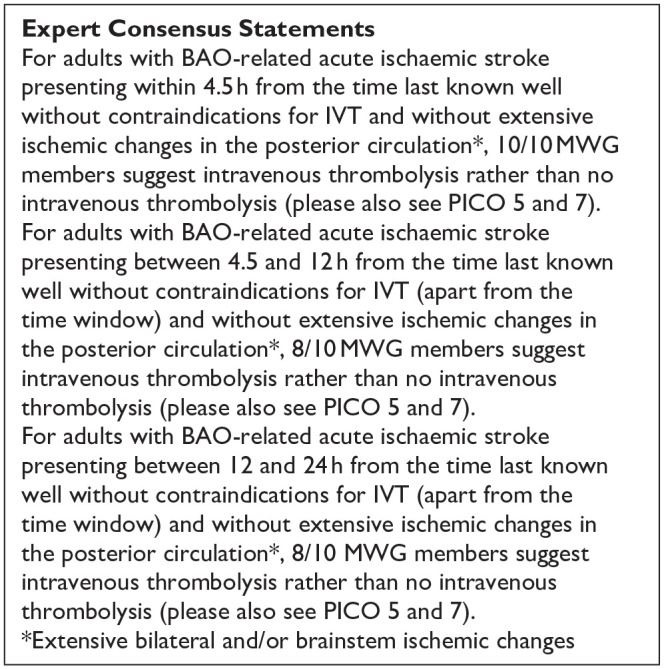


### PICO 2

For adults with BAO-related acute ischaemic stroke within 6 h of symptoms onset, does endovascular treatment (EVT) plus BMT compared with BMT alone improve outcomes?

### Analysis of current evidence

The literature search identified three RCTs addressing this PICO question. Only one trial recruited patients within 6 h of estimated symptom onset, while the other two recruited patients within 8 and 12 h.

BASICS (Endovascular Therapy for stroke due to Basilar-Artery Occlusion) was a multicentre, international, open-label with blinded outcome assessment RCT of EVT for BAO conducted at 23 centres in seven countries.^7^ Patients were randomised in a 1:1 ratio within 6 h of the estimated time of onset to receive EVT (intervention) or BMT (control), which was IVT in 80% of patients.^7^ At the beginning of recruitment, patients were eligible if they were younger than 85 years of age and had an NIHSS score of 10 or more. After the inclusion of 91 patients, inclusion criteria were expanded to allow recruitment of patients, who were 85 years of age or older, those who had an NIHSS score of less than 10, and those who had contraindications to IVT. The primary outcome was a favourable functional outcome, defined as a mRS score of 0–3. A total of 300 patients were enrolled (154 in the EVT group and 146 in the BMT group). There was no difference in the proportion of patients with a good outcome (mRS 0–3 at 3 months: 44% EVT vs. 38% BMT, RR 1.18, 95% CI: 0.92–1.50), favourable outcome (mRS 0–2) or distribution of mRS scores. sICH occurred in 4.5% of patients after EVT and in 0.7% of those after BMT (RR, 6.9; 95% CI: 0.9–53.0).

BEST (Endovascular Treatment versus standard medical treatment for vertebrobasilar artery occlusion) was a multicentre, prospective, open label with blinded outcome assessment RCT of EVT for vertebrobasilar occlusion at 28 centres in China (NCT02441556).^6^ Patients were randomised in a 1:1 ratio within 8 h of the angiography-confirmed BAO to receive EVT (intervention group) or BMT (control group), which included IVT in only 30% of patients. Patients were eligible if they were 18 years of age or older, had an occlusion of the basilar artery or the distal intracranial vertebral artery with no flow to the basilar artery. The primary outcome was favourable functional outcome defined as a mRS score of 0–3 at 3 months. The trial was terminated early after enrolling 131 patients (66 in the EVT group and 65 in the BMT group), because of excessive crossovers and a progressive drop in the rate of recruitment. The median NIHSS at baseline was very high, 32 in the EVT and 26 in the standard arm. There was a substantial rate of crossovers (22.5% from the BMT arm into EVT), and no difference in the proportion of patients with a good outcome (mRS 0–3 at 3 months: 42% EVT vs. 32% control, adjusted RR, 1.74, 95% CI: 0.81–3.74).

ATTENTION (Endovascular Treatment for Acute Basilar-Artery Occlusion) was a multicentre, prospective, open-label RCT of EVT for BAO at 36 centres in China.^8^ Patients were randomised in a 2:1 ratio within 12 h (median time from onset to randomisation was 5 h (3.5–7.0)) after the estimated time of onset to receive EVT (intervention) or BMT (control), which was IVT in only every third patient. Patients were eligible if they were at least 18 years of age and had NIHSS ⩾ 10. Furthermore, for patients <80 years of age, a pc-ASPECTS of at least 6 was required, whereas for those older than 80, it was at least 8. The estimated time of occlusion occurrence was defined as a sudden onset of BAO symptoms, with no consideration of any preceding minor prodromal symptoms. For patients with unknown time of stroke onset, a 12-h time window was calculated from the last time the patient was seen well. The primary outcome was good functional outcome defined as a mRS score of 0–3 at 3 months. A total of 340 patients were included in the intention-to-treat analysis: 216 and 124 patients were randomised within and beyond 6 h from symptom onset, respectively. EVT was associated with a higher proportion of patients with good outcomes (mRS 0–3 at 3 months) compared to BMT (46% vs. 23%, adjusted rate ratio 2.06 and 95% CI: 1.46–2.91; *p* < 0.001).

**Figure 2.1.**
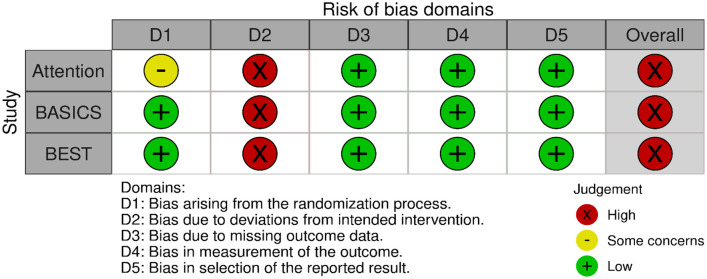
PICO 2 – Risk of bias for RCTs included in PICO 2.

All three trials presented performance bias, as the randomised participants and the treating physicians were aware of the allocated intervention (Figure 2.1). Furthermore, minor deviations from the intended interventions were noted in two RCTs. In addition, the ATTENTION trial did not clearly report the use of a minimization process to balance the two treatment groups with appropriate stratification, leading to some concerns about randomisation bias. In the BEST trial, a high rate of crossover occurred, and the final sample size was only 38% of the planned target of 344 patients, resulting in an underpowered analysis. Furthermore, there may have been a selection bias, as one third of patients declined trial participation. Regarding indirectness, the BEST trial included patients with very severe symptoms (median NIHSS 32), while the ATTENTION trial included patients with at least 10 NIHSS points. In contrast, BASICS trial started with patients having NIHSS ⩾ 10, but the inclusion criteria were later modified to include the whole range of NIHSS scores. Furthermore, controls are not directly comparable between the three trials, because the proportion of IVT in BMT and timing of IVT administration differed significantly among the trials. Only the BASICS trial included patients with a time window of 6 h, whereas in the BEST and ATTENTION trials the time window was 8 and 12 h, respectively. However, there are remarkable differences in the definition of time windows among the trials.

**Figure 2.2.**
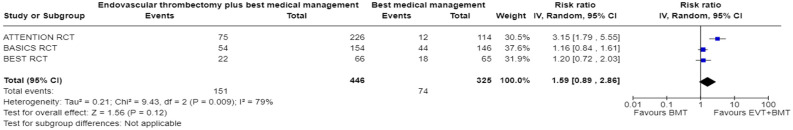
PICO 2 – Meta-analysis of randomised-controlled clinical trials: favourable functional outcome (mRS scores of 0–2 at 3 months) in patients with acute ischaemic stroke presenting within 6 h from the time last known well, treated with endovascular treatment plus best medical treatment (BMT) versus BMT alone (pooled adjusted RR, random-effects meta-analysis, *p* = 0.12).

We conducted a random-effects meta-analysis of studies that reported outcomes deemed critical and important. Furthermore, for functional outcomes, we performed additional analyses to test for interactions among RCTs with high versus low percentages of IVT in the BMT arm of a study (Figures 2.2–2.8). The BEST trial was excluded from this interaction analysis due to its extremely high rate of crossovers (22.5%) from EVT into BMT arm.^6^ The ATTENTION investigators listed in the limitation section that initially, patients had to pay for the thrombolytic drug, which may have contributed to the low use of thrombolytics.^8,9^ We identified several significant interactions (see Table 1), further supported by the fact that no difference between EVT and BMT was observed in the BASICS trial,^7^ while in the ATTENTION trial,^8^ no superiority of EVT was observed in the analysis when BMT included 100% IVT (adjusted rate ratios 1.57 (95% CI: 0.97–2.54)). Frequencies of sICH were significantly higher in the EVT arms.

**Figure 2.3.**
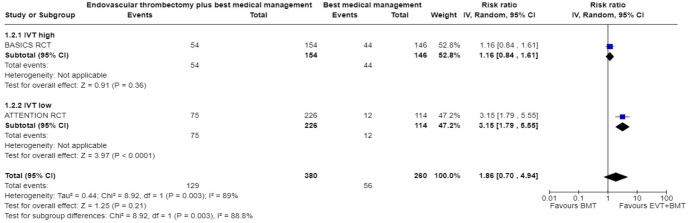
PICO 2 – Meta-analysis of randomised-controlled clinical trials: favourable functional outcome (mRS scores of 0–2 at 3 months) in patients with acute ischaemic stroke presenting within 6 h from the time last known well, treated with endovascular treatment plus best medical treatment (BMT) versus BMT alone, and stratified by high versus low proportion of IVT-treated patients in the BMT arm (pooled adjusted RR, random-effects meta-analysis, *p* = 0.003 for interaction). The BEST trial was excluded from this interaction analysis due to its extremely high rate of crossovers (22.5%) from EVT into BMT arm.

**Figure 2.4.**
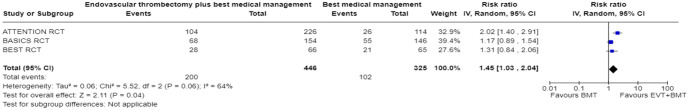
PICO 2 – Meta-analysis of randomised-controlled clinical trials: Good functional outcome (mRS scores of 0–3 at 3 moths) in patients with acute ischaemic stroke presenting within 6 h from the time last known well, treated with endovascular treatment plus best medical treatment (BMT) versus BMT alone (pooled adjusted RR, random-effects meta-analysis, *p* = 0.04).

**Figure 2.5.**
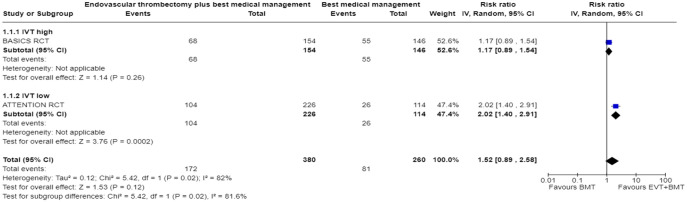
PICO 2 – Meta-analysis of randomised-controlled clinical trials: Good functional outcome (mRS scores of 0–3 at 3 months) in patients with acute ischaemic stroke presenting within 6 h from the time last known well, treated with endovascular treatment plus best medical treatment (BMT) versus BMT alone, and stratified by high versus low proportion of IVT-treated patients in the BMT arm (pooled adjusted RR, random-effects meta-analysis, *p* = 0.02 for interaction). The BEST trial was excluded from this interaction analysis due to its extremely high rate of crossovers (22.5%) from EVT into BMT arm.

**Figure 2.6.**
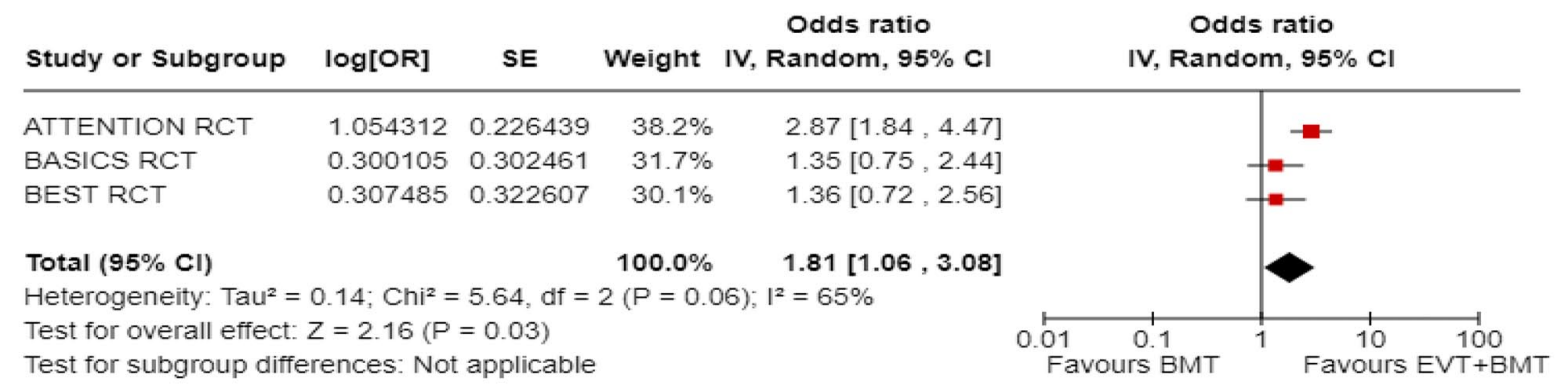
PICO 2 – Meta-analysis of randomised-controlled clinical trials: Distribution of mRS scores at 3 months (shift analysis) in patients with acute ischaemic stroke presenting within 6 h from the time last known well, treated with endovascular treatment plus best medical treatment (BMT) versus BMT alone (pooled adjusted RR, random-effects meta-analysis, *p* = 0.03).

**Figure 2.7.**
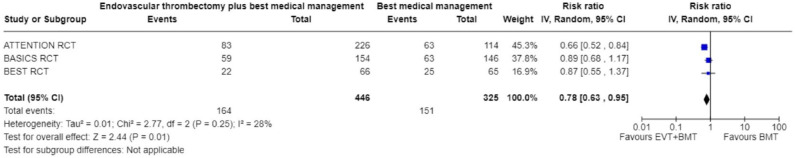
PICO 2 – Meta-analysis of randomised-controlled clinical trials: mortality at 90 days in patients with acute ischaemic stroke presenting within 6 h from the time last known well, treated with endovascular treatment plus best medical treatment (BMT) versus BMT alone (pooled adjusted RR, random-effects meta-analysis, *p* = 0.01).

**Figure 2.8.**
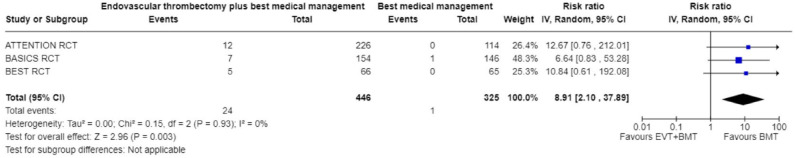
PICO 2 – Meta-analysis of randomised-controlled clinical trials: symptomatic ICH in patients with acute ischaemic stroke presenting within 6 h from the time last known well, treated with endovascular treatment plus best medical treatment (BMT) versus BMT alone (pooled adjusted RR, random-effects meta-analysis, *p* = 0.003).

**Table 1.** GRADE evidence profile for PICO 2.

Certainty assessment							No. of patients		Effect		Certainty	Importance
No. of studies	Study design	Risk of bias	Inconsistency	Indirectness	Imprecision	Other considerations	EVT plus BMT	BMT alone	Relative (95% CI)	Absolute (95% CI)		
mRS 0–3 at 90 days: RCT												
3	Randomised trials	Serious^a^	Serious^b^	Serious^c,d,e^	Serious^f^	None	200/446 (44.8%)	102/325 (31.4%)	RR 1.45 (1.03–2.04)	141 more per 1000 (from 9 more to 326 more)	⨁○○○	Critical
											Very low	
mRS 0–2 at 90 days: RCT												
3	Randomised trials	Serious^a^	Serious^g^	Serious^c,d,e^	Serious^f^	None	151/446 (33.9%)	74/325 (22.8%)	RR 1.59 (0.89–2.86)	134 more per 1000 (from 25 fewer to 424 more)	⨁○○○	Critical
											Very low**	
Shift (ordinal) mRS at 90 days: RCT												
3	Randomised trials	Serious^a^	Serious^h^	Serious^c,d,e^	Serious^f^	None	–	–	OR 1.81 (1.06–3.08)	2 fewer per 1000 (from 3 fewer to 1 fewer)	⨁○○○	Critical
											Very low***	
Mortality at 90 days: RCT												
3	Randomised trials	Serious^a^	Serious^a^	Serious^c,d,e^	Serious^f^	None	164/446 (36.8%)	151/325 (46.5%)	RR 0.78 (0.63–0.95)	102 fewer per 1000 (from 172 fewer to 23 fewer)	⨁○○○	Important
											Very low	
Symptomatic intracranial haemorrhage (sICH): RCT												
3	Randomised trials	Serious^a^	Not serious	Serious^c,d,e^	Serious^f^	Very strong association	24/446 (5.4%)	1/325 (0.3%)	RR 8.91 (2.10–37.89)	24 more per 1000 (from 3 more to 114 more)	⨁⨁⨁○	Important
											Moderate	
mRS 0–3 NRSI												
3	Non-randomised studies	Serious^i^	Serious^j^	Serious^k^	Serious^l^	None	–	–	RR 0.86 (0.47–1.59)	1 fewer per 1000 (from 2 fewer to 0 fewer)	⨁○○○	Critical
											Very low****	

CI: confidence interval; OR: odds ratio; RR: risk ratio.

a Serious risk of bias arising from the deviations from intended intervention in all RCTs, high risk of performance bias. Some concerns in other domains.

b *I*^2^ statistic, which quantifies the proportion of the variation in point estimates due to among-study differences was 64%, assessed as substantially high.

c Enrolled patients had severe/very severe symptoms. Patients with mild-to-moderate symptoms were missing or underrepresented.

d Comparator not the same in the trials; it differs by proportion of IVT in the BMT arms and by timing of IVT administration.

e Time window 6 h only in 1 trial, whereas 8 and 12 h in the other 2 trials.

f Serious imprecision due to low optimal information size. The total number of patients included is less than the number of patients generated by a conventional size sample calculation for a single adequately powered clinical trial.

g *I*^2^ statistic, which quantifies the proportion of the variation in point estimates due to among-study differences was 79%, assessed as substantially high.

h *I*^2^ statistic, which quantifies the proportion of the variation in point estimates due to among-study differences was 65%, assessed as substantially high.

i Serious risk of bias due to serious confounding reported in one of these studies implemented for this outcome according to ROBINS-I tool for observational studies.

j Presence of heterogeneity.

k Raw data was not available in one study; generic inverse variance meta-analysis of the reported RRs in the studies was performed.

l Inconclusive confidence interval.

* *p*-value for interaction between trials with high (European trial) and low (Asian trials) proportion of IVT in BMT arms (0.02).

** *p*-value for interaction between trials with high (European trial) and low (Asian trials) proportion of IVT in BMT arms (0.003).

*** *p*-value for interaction between trials with high (European trial) and low (Asian trials) proportion of IVT in BMT arms (0.03).

**** *p*-value for interaction between registry studies with high (European) and low (Asian) proportion of IVT in BMT arms (<0.00001).

### Additional information

**Figure 2.9.**
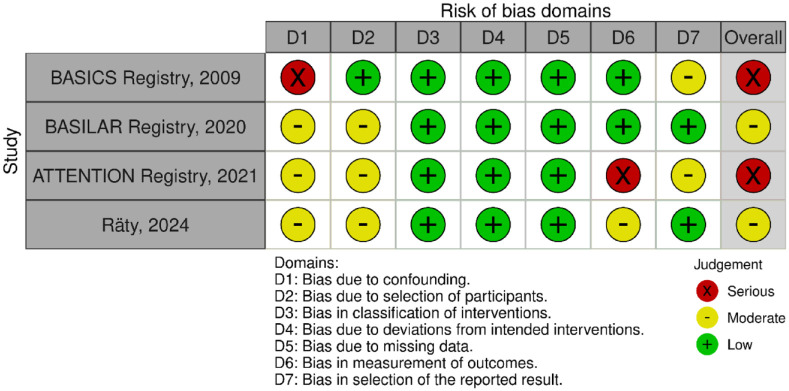
PICO 2 – Risk of bias for registry studies.

The literature search identified three registry-based non-randomised studies addressing this PICO question, the bias of which is described in Figure 2.9 and in PICO 3.

The BASILAR (Endovascular treatment for Acute Basilar Artery Occlusion Study) registry was a nationwide prospective registry of consecutive patients presenting with an acute, symptomatic, radiologically confirmed BAO at 47 comprehensive stroke centres across 15 provinces in China between January 2014 and May 2019.^26^ Patients with BAO within 24 h of estimated symptom onset were divided into groups receiving BMT plus EVT (*n* = 647) or BMT alone (*n* = 182), of whom 463 and 127 were treated within 6 hours from symptom onset, respectively. The rate of IVT in the whole cohort was 20%. The primary clinical outcome was the improvement in mRS scores at 3 months across the two treatment groups assessed as a common odds ratio using ordinal logistic regression shift analysis, adjusted for prespecified prognostic factors. The secondary efficacy clinical outcome was good functional status, defined as mRS scores of 0–3 at 3 months. However, the only reported outcome for the 6-h time window is distribution of mRS at 3 months (common odds ratio).

The BASICS registry^2^ was a prospective, international (Europe, South America, North America, Australia), observational registry of consecutive patients who presented with an acute symptomatic and radiologically confirmed BAO between 1 November 2002 and 1 October 2007. The primary clinical outcome was assessed at 1 month and defined as mRS scores of 4–6. Patients presenting within 24 h from symptom onset were divided into three groups according to the treatment they received: antithrombotic treatment only (AT), which comprised antiplatelet drugs or systemic anticoagulation; primary intravenous thrombolysis (IVT), including subsequent intra-arterial thrombolysis; or intra-arterial therapy (IAT), which comprised intra-arterial thrombolysis, mechanical thrombectomy, stenting, or a combination of these approaches. Of the 592 patients who were analysed, 183 were treated with only AT, 121 with IVT, and 288 with IAT. A total of 84, 99 and 186 within 6 h, respectively. The patient-level outcome data (unadjusted mRS 0–3) for the 6-h time window are available only for IVT and IAT subgroups.

The ATTENTION registry^27^ is an ongoing prospective, multicentre registry in China. The sample comprised 2134 patients within 24 h of estimated time of acute BAO recruited at 48 comprehensive stroke centres between March 2017 and February 2021. Four hundred sixty-two patients received BMT (less than 20% IVT) and 1672 underwent EVT plus BMT. The median time from estimated time of BAO to treatment was 419 min (IQR: 273–682), but the number of patients treated with BMT as well as the combination of EVT with BMT within 6 h from symptom onset was unavailable in the relevant publication. BMT consisted of IVT, antiplatelets, anticoagulants or combinations. Endovascular approach consisted of mechanical thrombectomy, thromboaspiration, stenting, IA thrombolysis or combination. The primary clinical outcome was a favourable functional outcome, defined as mRS scores of 0–3 at 3 months. The outcome data were reported as RR, and the number of the patients in the subgroups was not reported. All other studies reported either raw data or odds ratios.

The registry study by Räty et al.,^25^ compared 122 of IVT-only versus EVT ± IVT treated BAO patients. The primary outcome was mRS 0–3 and the data were analysed with conventional and doubly robust inverse probability-weighted regression analysis. The primary outcome was more frequent in IVT only group compared to EVT ± IVT. In that study, about 60% of patients had delays of less than 6 h.

**Figure 2.10.**
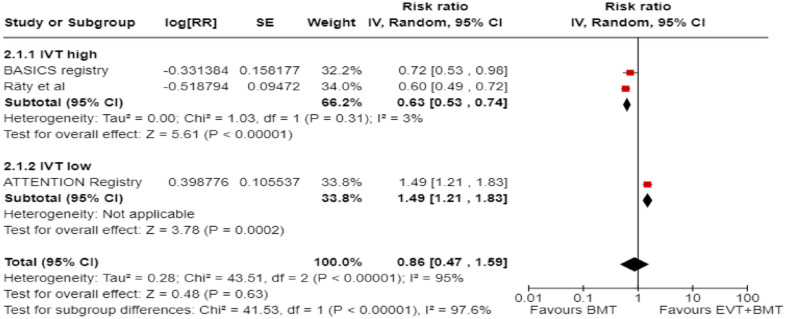
PICO 2 – Meta-analysis of registry studies: Good functional outcome (mRS scores 0–3 at 3 months) in patients with acute ischaemic stroke presenting within 6 h from the time last known well, treated with endovascular treatment plus best medical treatment (BMT) versus BMT alone, and stratified by high versus low proportion of IVT-treated patients in the BMT arm (pooled adjusted RR, random-effects meta-analysis, *p* = 0.0001 for interaction).

**Figure 2.11.**
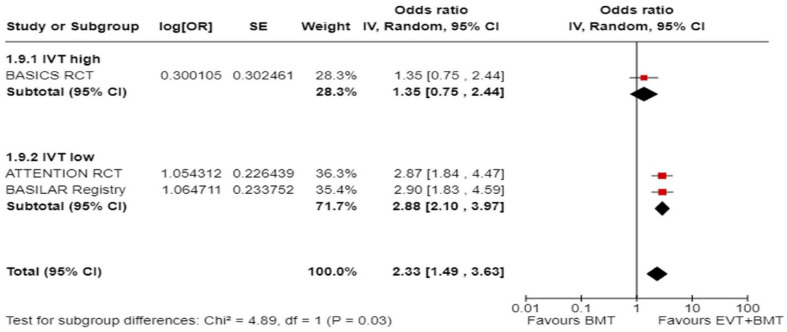
PICO 2 – Forest plot showing differential effect of reperfusion therapy stratified by high versus low proportion of IVT-treated patients in the BMT arm (*p* = 0.03 for interaction), including data from randomised-controlled clinical trials (RCTs) and one registry study. Distribution of mRS scores at 3 months (shift analysis) in patients with acute ischaemic stroke presenting within 6 h from the time last known well, treated with endovascular treatment plus best medical treatment (BMT) versus BMT alone (Cochran’s Q-test for interaction testing).

Differential effect of reperfusion therapy stratified by high versus low proportion of IVT-treated patients in the BMT arm is outlined in Figures 2.10 and 2.11.

Table 1 provides details regarding the assessment of the quality of evidence for all outcomes evaluated in PICO 2. To better understand the differential effect of reperfusion therapy stratified by the composition of BMT, please see also PICO 3 and the discussion.


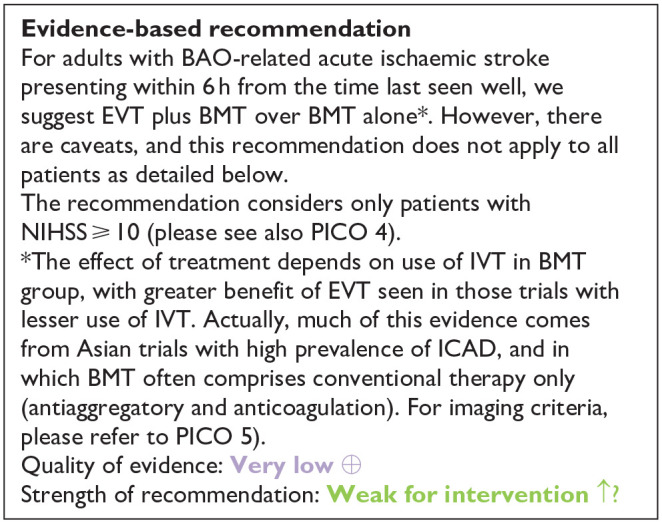


### PICO 3

For adults with BAO-related acute ischaemic stroke 6–24 h from time last known well, does EVT plus BMT compared with BMT alone improve outcomes?

### Analysis of current evidence

The literature search identified two published RCTs^8,9^ addressing this PICO question.

The ATTENTION trial was otherwise described in PICO 2, however, we want to point out that only one patient received IVT in the time window of more than 6 h from estimated time of BAO to imaging. BAOCHE (Basilar Artery Occlusion Chinese Endovascular) trial, a multicentre Chinese prospective RCT, aimed to assess the effect and safety of EVT in conjunction with BMT compared with BMT alone. The trial enrolled patients with AIS due to BAO and an absence of large baseline infarct on neuroimaging who underwent randomisation in 6–24 h after symptom onset.^9^ Symptoms onset was defined as a time point when symptoms started or, if unknown, as time when patients were last seen well. Isolated vertigo was not considered onset of symptoms. Treatment start was defined as time of groin puncture. The original primary outcome, a mRS score of 0–4 at 3 months, was subsequently changed to a good functional status (mRS-scores of 0–3).

**Figure 3.1.**
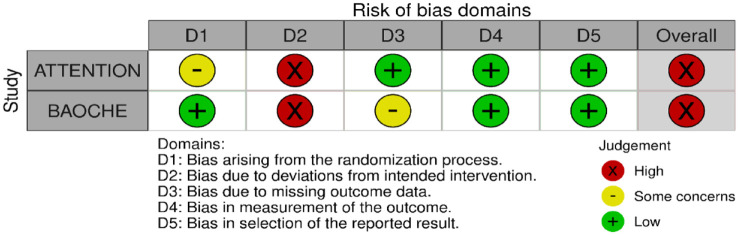
PICO 3 – Risk of bias in randomised-controlled clinical trials.

Assessment of the risk of bias is presented in Figure 3.1.

Both trials^8,9^ presented performance bias, as randomised participants and treating physicians were aware of the allocated intervention. Furthermore, minor deviations from the intended interventions were noted in both RCTs. In addition, the ATTENTION trial^8^ did not clearly report the use of a minimization process to balance the two treatment groups with appropriate stratification, leading to some concerns about randomisation bias. Finally, the BAOCHE trial^9^ presented minor concerns due to missing outcome data. The overall risk of bias was high both for ATTENTION^8^ and BAOCHE^9^ trials.

**Figure 3.2.**
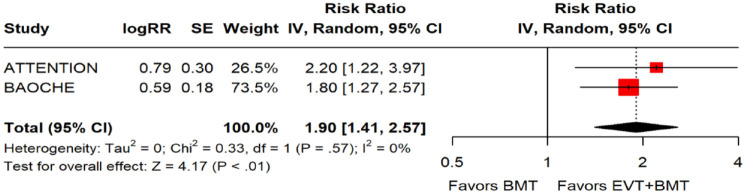
PICO 3 – Meta-analysis of randomised-controlled clinical trials (RCTs): Good functional outcome (mRS scores 0–3 at 3 months) in patients with acute ischaemic stroke presenting within 6–24 h from time last known well, treated with endovascular treatment plus best medical treatment (BMT) versus BMT alone (pooled adjusted RR, random-effects meta-analysis).

Data regarding patients presenting within 6–24 h from time last known well were available in one of the trials only as adjusted RRs with corresponding 95% CIs, without presenting the raw data. For that reason, we used a generic inverse variance meta-analysis to provide a pooled overall effect (Figure 3.2). Compared to patients randomised to BMT, the pooled adjusted RR for a good functional outcome in patients randomised to EVT was 1.90 (95% CI: 1.41–2.57; *p* < 0.01; *I*^2^: 0%; Figure 3.2).

### Additional information

The literature search identified four registry-based observational studies addressing this PICO question.^2,26–28^ The ATTENTION registry^27^ was described in PICO 2. Qualifying patients had to present within 24 h of estimated symptom onset. The number of patients treated with BMT as well as the combination of EVT with BMT beyond 6 h from symptom onset was unavailable in the relevant publication. The BASILAR registry,^26^ a nationwide prospective registry, was described in PICO 2. A total of 184 and 55 patients were treated with BMT plus EVT and BMT alone beyond 6 hours from symptom onset, respectively. The BASICS registry^2^ was described in PICO 2. A total of 99, 21 and 102 patients received AT, IVT and IAT beyond 6 h, respectively.

A registry presented by Gruber et al.^28^ was a mandatory prospective stroke inpatient quality assurance registry covering the entire federal state of Hessen in Germany. Gruber et al.^28^ analysed the clinical course and short-term outcomes of patients with radiologically confirmed acute BAO dichotomised by BMT plus EVT (*n* = 270) or BMT alone (*n* = 133). This registry also included patients presenting beyond 24 h from symptom onset (*n* = 26) and with unknown time from symptom onset (*n* = 58). The primary clinical outcome was good functional status, defined as mRS score of 0–3 at 3 months. A total of 46 and 30 patients were treated with BMT plus EVT and BMT alone between 6 and 24 h from symptom onset, respectively.

The registry study by Räty et al.^25^ was described in PICO 2. It compared 122 of IVT-only vs EVT ± IVT treated BAO patients and included about 40% of patients with delays of more than 6 h.

**Figure 3.3.**
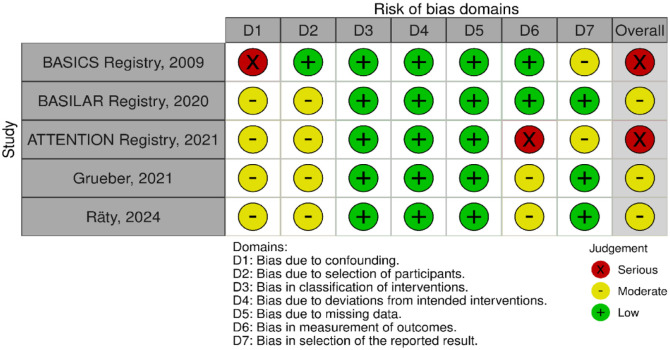
PICO 3 – Risk of bias in observational studies.

The MWG assessment of the risk of bias in the included observational studies for PICO 3 was performed according to the Cochrane ROBINS-I tool^10^ and is presented in Figure 3.3.

All four studies presented moderate confounding bias, since there were several significant baseline differences between the different treatment groups. The ATTENTION registry,^27^ the BASILAR registry,^26^ the registry presented by Gruber et al.,^28^ and by Räty et al.,^25^ were based on data derived from centres of specific countries (i.e. China in the first two studies, Germany in the third, and Finland in the last), thus moderate selection bias may occur. No significant misclassification, deviation from intervention, or missing bias occurred in any of the included observational studies. Assessment by blinded, certified investigators was reported to have been performed only in the BASILAR registry, while in the other three studies no clear description of the assessment was presented. The BASICS registry^2^ did not predefine sICH as an outcome measure, and the follow-up period was restricted to only 1 month, rendering the study vulnerable to serious reporting bias. Finally, the study of Gruber et al.^28^ presents moderate reporting bias since sICH was not assessed or reported as a safety outcome.

**Figure 3.4.**
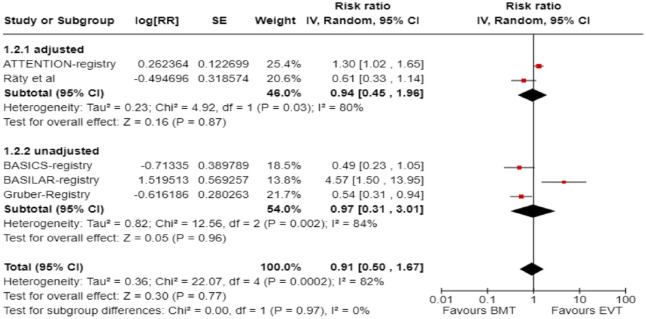
PICO 3 – Meta-analysis of observational studies: Good functional outcome (mRS scores 0–3 at 3 months, except for the BASICS registry: 1 month) in patients with acute ischaemic stroke presenting within 6–24 h from time last known well, treated with endovascular treatment plus best medical treatment (BMT) versus BMT alone (pooled RR, random-effects meta-analysis).

We conducted a study-level, random-effects meta-analysis of the four observational studies included in PICO 3 for the outcome mRS score of 0–3 at 3 months. However, it should be noted that the ATTENTION registry reported only the adjusted RR for the patients presenting within 6–24 h from time last -known well and achieving mRS 0–3 at 3 months, without providing raw data. Therefore, we were not able to calculate the unadjusted RR for this study. We used the generic inverse variance meta-analysis to provide a pooled overall effect, but we also presented two subgroups stratifying by the adjusted versus unadjusted RR. Patients treated with EVT had a similar likelihood of achieving mRS 0–3 at 3 months compared to patients treated with BMT (Figure 3.4).

**Figure 3.5.**
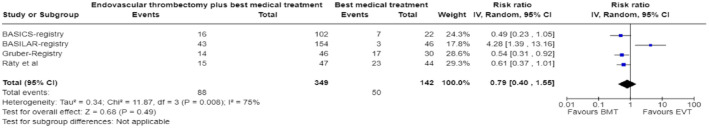
PICO 3 – Sensitivity analysis of observational studies after inclusion of the studies that presented raw data regarding good functional outcome (mRS scores 0–3 at 3 months, except for the BASICS registry: 1 month) in patients with acute ischaemic stroke presenting within 6–24 h from time last known well, treated with endovascular treatment plus best medical treatment (BMT) versus BMT alone (RR, random-effects meta-analysis).

A sensitivity analysis was performed by including only the four studies that presented raw data, and similar results were obtained (Figure 3.5).

**Figure 3.6.**
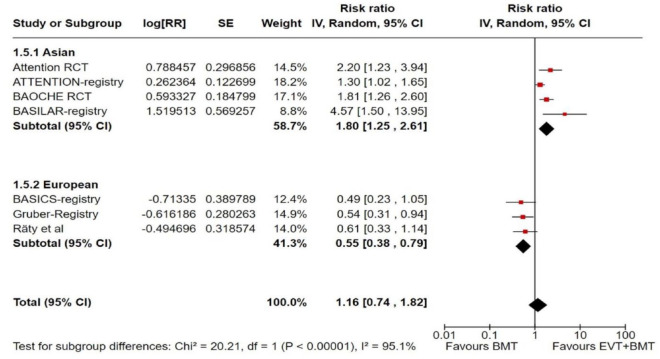
PICO 3 – Forest plot showing differential effect of reperfusion therapy stratified by geographical regions including RCTs and observational studies: Good functional outcome (mRS scores 0–3 at 3 months, except for the BASICS registry: 1 month) in patients with acute ischaemic stroke presenting within 6–24 h from time last known well treated with endovascular treatment plus best medical treatment (BMT) versus BMT alone (*p*-value for interaction <0.0001, Cochran’s Q-test for interaction testing).

Further, we present forest plot showing differential effect of reperfusion therapy stratified by geographical region, in which the patients were randomised (Asian vs. European/International) (Figure 3.6). In line with the findings presented in PICO 2, we found a significant interaction (*p* < 0.00001) between the two regions. In the Asian studies, EVT led to better outcomes compared to BMT, whereas the opposite trend was observed in the European/International studies. There are several plausible explanations for this heterogeneity, including differences in systems of care and ethnicity-related issues.

The BAOCHE and ATTENTION investigators listed in the limitation section that initially, patients had to pay for the thrombolytic drug, which may have contributed to the low use of thrombolytics.^8,9^ Notably, in both the ATTENTION and BAOCHE trials, no superiority of EVT was observed in analysis when BMT included 100% IVT (adjusted rate ratios 1.57 (95% CI: 0.97–2.54) and 1.74 (95% CI: 0.36–8.4), respectively).^8,9^

It is not known how standard treatment differs among various centres worldwide for patients who underwent EVT compared to those who have not received any reperfusion therapy at all (as was the case in most of the patients in Asian trials, who received merely secondary prevention). It is possible that the latter group was not admitted to intensive or intermediate care units. Regarding ethnicity-related issues, the high prevalence of ICAD in the Asian population was mentioned as a reason why the results of the BAOCHE and ATTENTION trials may not be generalizable to Western countries.^8,9^ Finally, the ATTENTION investigators acknowledged that their results are not generalizable to patients with an NIHSS of less than 10.^8,9^

Table 2 provides details regarding the assessment of the quality of evidence for all outcomes evaluated in PICO 3 both using randomised and observational data.


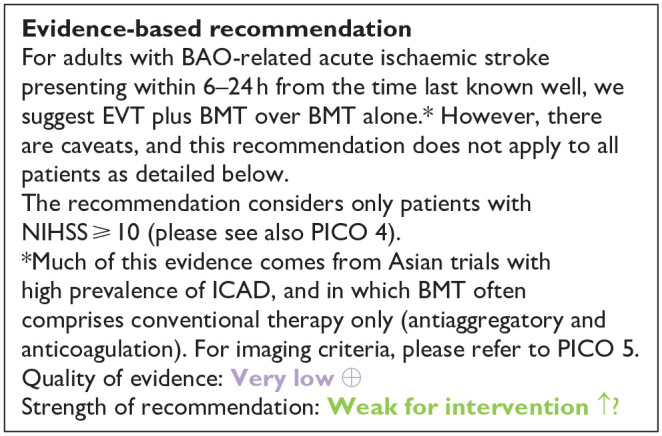


**Table 2.** GRADE evidence profile for PICO 3.

Certainty assessment							No. of patients		Effect		Certainty	Importance
No.	Study design	Risk of bias	Inconsistency	Indirectness	Imprecision	Other	EVT plus BMT	BMT alone	Relative (95% CI)	Absolute (95% CI)		
mRS 0–3 at 90 days												
2	Randomised trials	Serious^a^	Not serious	Serious^c h^	Not serious	None	NA	NA	aRR: 1.90 (1.41–2.57)	NA	⨁⨁○○	Critical
											Low	
5	Prospective registries	Serious^b^	Serious^d^	Serious^c^	Serious^e^	None	NA	NA	RR: 0.91 (0.50–1.67)	NA	⨁○○○	Critical
											Very low	
mRS 0–2 at 90 days												
1	Randomised trial	Serious^a^	NA^f^	Serious^h^	Not serious	None	43/110	15/107	aRR 2.75 (1.65–4.56)	25 more per 100 (from 14 to 36 more)	⨁○○○	Critical
											Very low	
1	Prospective registry	Moderate^b^	NA^f^	Not serious	Serious^g^	None	36/154	1/46	RR 10.75 (1.52–76.31)	21 more per 100 (from 13 to 29 more)	⨁○○○	Critical
											Very low	
Shift (ordinal) mRS at 90 days												
1	Randomised trial	Serious^a^	NA^f^	Serious^h^	Not serious	None	NA/110	NA/107	acOR 2.64 (1.54–4.50)	NA	⨁○○○	Critical
											Very low	
1	Prospective registry	Moderate^b^	NA^f^	Not serious	Serious^g^	None	NA	NA	cOR 4.1 (1.8–9.5)	NA	⨁○○○	Critical
											Very low	
Mortality at 90 days												
1	Randomised trial	Serious^a^	NA^f^	Serious^h^	Serious^e^	None	34/110	45/107	aRR 0.75 (0.54–1.04)	11 fewer per 100 (from 24 fewer to 2 more)	⨁○○○	Critical
											Very low	
1	Prospective registry	Moderate^b^	NA^f^	Not serious	Not serious	None	72/154	34/46	RR 0.63 (0.50–0.80)	27 fewer per 100 (42 to 12 fewer)	⨁○○○	Critical
											Very low	
Symptomatic intracranial haemorrhage (sICH)												
1	Randomised trial	Serious^a^	NA^f^	Serious^h^	Very seriouse^e,g^	None	6/102	1/88	RR 5.18 (0.64–42.18)	5 more per 100 (0–10 more)	⨁○○○	Critical
											Very low	
mTICI (TICI: thrombolysis in cerebral ischemia)												
1	Randomised trial	Serious^a^	NA^f^	Serious^h^	NA	None	89/101	NA	NA	NA	⨁○○○	Important
											Very low	

a Serious risk of bias arising from the deviations from intended intervention in all RCTs, high risk of performance bias. Some concerns in other domains.

b Moderate risk of confounding and selection bias.

c Raw data was not available in one study; generic inverse variance meta-analysis of the reported RRs in the studies was performed.

d Presence of heterogeneity.

e Inconclusive confidence interval.

f Only one study included. Evaluation of inconsistency is not applicable (NA).

g Wide confidence interval.

h Enrolled patients had mostly severe/very severe symptoms. Patients with mild-to-moderate symptoms were missing or underrepresented.

### PICO 4

For adults with BAO-related acute ischemic stroke, does selection of reperfusion treatment (IVT or EVT) based on specific presentation (e.g. high NIHSS cutoff, coma on admission, proximal location of basilar artery occlusion) compared with other presentation features (e.g. low NIHSS cutoff, no coma on admission, distal location of basilar artery occlusion) modify the outcome?

### Analysis of current evidence

The aim of this PICO question was to investigate the presence or absence of a difference in treatment effect (interaction/effect modification) based on a specific presentation (i.e. severity of neurological symptoms and/or occlusion location) at baseline. To address this question, we focused on reperfusion therapy studies that provide subgroup analyses stratified by a specific baseline situation. For the comparison of EVT (±IVT) versus no EVT, the literature search identified four RCTs and three registries that reported outcomes at 3 months.^2,6,8,9,26,27,29^

One observational study, which reported outcomes only at 1 month,^2^ is described in additional information section.

#### EVT versus no EVT depending on initial stroke severity

The four identified RCTs, BEST, BASICS, ATTENTION, and BAOCHE, have all been described in PICO questions 2 and 3. All trials reported subgroup analyses stratified by baseline NIHSS score, but the stratification cutoff differed substantially across the trials. Some of the NIHSS cut-off values are of lesser clinical relevance (29 in BEST and 20 in BAOCHE and in ATTENTION). In the BEST trial, there was no evidence of a differential effect (*p* for interaction = 0.79) of EVT versus no EVT on mRS 0–3 at 90 days in patients with NIHSS score ⩽ 29 (OR: 1.56; 95% CI: 0.60–4.10) and >29 (OR: 1.91; 95% CI: 0.61–6.00). In the ATTENTION trial, the adjusted RR for the association between EVT and mRS 0–3 at 3 months were 1.51 (1.05–2.18) and 3.53 (1.71–7.29) in patients with NIHSS score 10–19 and ⩾20, respectively. No *p*-value for interaction was reported. No data exist for less than 10 NIHSS points, because inclusion criteria in ATTENTION was 10 or higher. In the BASICS trial, the RR for the association between EVT and mRS 0–3 at 3 months in patients with NIHSS score <10, 10–19 and ⩾20 were 0.85 (0.62–1.16), 1.55 (1.06–2.27), and 1.28 (0.67–2.46), respectively. No *p*-value for interaction was reported in the original publication, however, it was presented by Dr W. Schonewille during the ESOC 2020 and ESOC 2023 conferences: p-value for interaction was 0.02 and the conclusion was that EVT is not better than BMT in patients with BAO and less than 10 NIHSS points. We also performed a post-hoc interaction test, based on the data from the original publication of the BASICS trial and found very similar p-value for the interaction. Of note, BASICS was the only trial with high proportion (~80%) of IVT in the BMT arm. In the BAOCHE trial, the magnitude of the treatment effect on mRS 0–3 seemed similar in patients with NIHSS score 6–20 (adjusted RR 1.80 (1.21–2.67)) and >20 (adjusted RR 1.83 (0.73–4.58)). No *p*-value for interaction was reported in the original publication. However, very recent meta-analysis of the BASICS and BAOCHE trials^30^ reported outcomes of patients with BAO and NIHSS < 10. In this subgroup analysis of 78 patients, frequencies of favourable (mRS 0–3) or excellent (mRS 0–2) clinical outcome between the EVT and the BMT groups were comparable. Favourable functional outcome (mRS 0–3) at 3 months was achieved in 26 of 37 patients (70.3%) in the EVT group and in 30 of 41 patients (73.2%) in the BMT group. Excellent clinical outcome (mRS 0–2) occurred in 22 of 37 patients (59.5%) in the EVT group, and 24 of 41 patients (58.5%) in the BMT group. The rate of sICH in patients with NIHSS <10 was 8.1% in the EVT group, whereas no sICH occurred in the BMT group. The mortality rate in the EVT group was 18.9% (7 of 37 patients) and 17.1% (7 of 41) in the BMT group. *p*-value for the interaction for the primary outcome (mRS 0–3) was 0.04. Hence, in BAO patients with less than 10 NIHSS points, EVT is not superior to BMT and is less safe. The interaction (*p*-value) in subgroup analysis stratified by 10 NIHSS points was slightly different between the aforementioned meta-analysis BASICS and BAOCHE (p-value for interaction 0.04) compared to data from the BASICS trial alone (*p*-value for interaction 0.02). This difference may be explained by different proportion of IVT in the BMT arm of BASICS compared to BAOCHE (80% vs. 22%).

The BASILAR registry study was described in PICO 2 and 3. Only 20% of the patients received IVT (with alteplase or urokinase). Otherwise, BMT included antiplatelet drugs, systematic anticoagulation, or a combination of these treatments, at the discretion of the treating physician. Subgroup analyses according to a NIHSS cut-off of 26 points did not suggest a modification of treatment effect by baseline NIHSS score (adjusted common ORs for lower mRS scores at 90 days: 2.2, 95% CI: 1.3–3.6 in the NIHSS 0–26 subgroup; 3.3, 95% CI: 1.7–6.5 in the NIHSS > 26 subgroup; *P* for interaction = 0.52). Again, the selection of the cut-off value (NIHSS 26) is of lesser clinical relevance.

Between 2014 and 2016, 167 patients (median age: 75 (66–82); median NIHSS score: 24 (10–30)) were enrolled in the prospective multicentre RESCUE Japan Registry 2 study within 24 h of symptomatic BAO.^29^ The treatment applied was decided by the attending physician (EVT group, *n* = 129, 77.2% or BMT group, *n* = 38, 22.8%), and the analysis was stratified by baseline NIHSS score cut-off of 10 points. Proportion of patients who achieved mRS ⩽ 3 score at 3 months (primary outcome) after EVT compared with BMT (including IVT in about 24%) was 54% versus 12% (*p* < 0.01) in the severe subgroup (NIHSS score 10–40), and 72% versus 86% (*p* = 0.43) in the mild subgroup (NIHSS score 0–9). No *p*-value for interaction or adjusted analyses were provided in the original publication, however, we have computed *p*-value of 0.004 for this interaction.

The ATTENTION registry^27^ was described in PICO 2. The proportion of patients who achieved mRS ⩽ 3 score at 3 months (primary outcome) after EVT compared with BMT (including IVT in about 20%) was 36.8% versus 23.4% (adjusted relative risk 1.58 (95% CI: 1.30–1.91)) in the severe subgroup (NIHSS score at least 10), and 58.7% versus 51.4% (adjusted relative risk 1.05 (95% CI: 0.80–1.38)) in the mild subgroup (NIHSS score 0–9). Significant interaction was observed (*p* < 0.001).

**Figure 4.1.**
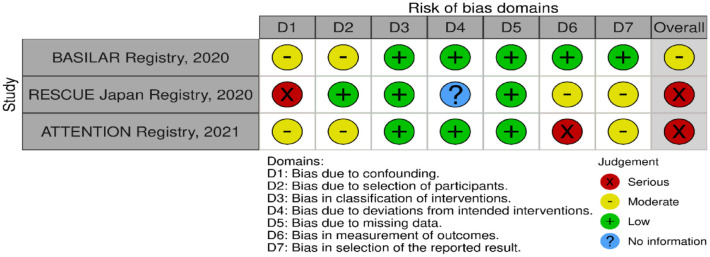
PICO 4 – Risk of bias in observational studies.

Evaluation of bias for the four RCTs is visualised in PICO 2 and 3, whereas bias for the three observational studies is in Figure 4.1.

**Figure 4.2.**
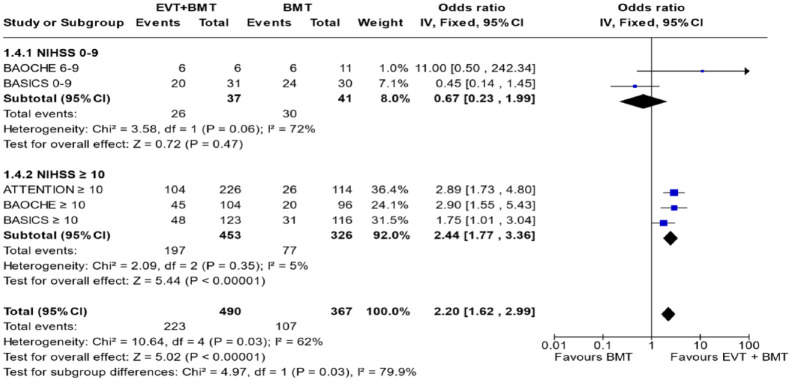
PICO 4 – Meta-analysis of randomised-controlled clinical trials (RCTs) stratified by clinical severity at baseline (*p*-value for interaction 0.03): Good functional outcome (mRS scores of 0–3 at 3 months) in patients with acute ischaemic stroke presenting within 6 h (BASICS), within 12 h (ATTENTION), or within 6–24 h (BAOCHE) from time last known well, treated with endovascular treatment plus best medical treatment (BMT) versus BMT alone (pooled RR, random-effects meta-analysis, Cochran’s Q-test for interaction testing). Only a minor proportion of patients randomised to ATTENTION and BAOCHE received IVT as part of the BMT.

**Figure 4.3.**
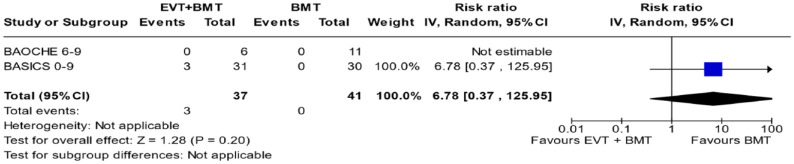
PICO 4 – Meta-analysis of randomised-controlled clinical trials (RCTs): Symptomatic intracranial haemorrhage in patients with acute ischaemic stroke presenting with <10 NIHSS, treated with endovascular treatment plus best medical treatment (BMT) versus BMT alone (RR, random-effects meta-analysis).

**Figure 4.4.**
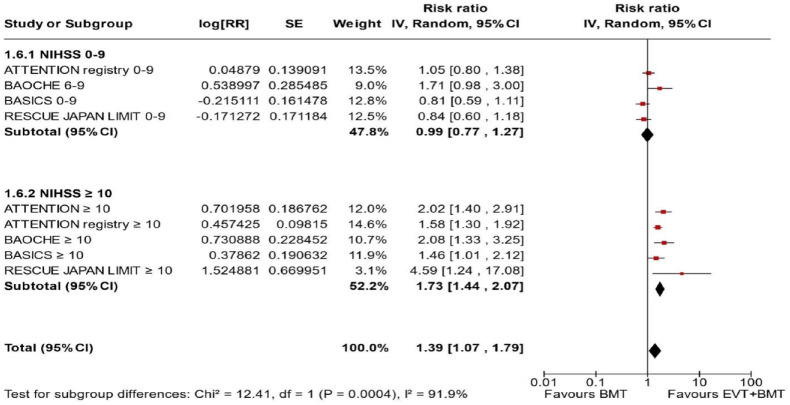
PICO 4 – Forest plot showing differential effect of reperfusion therapy stratified by NIHSS cutoff 10, including data from randomised-controlled clinical trials (RCTs) and registry studies. Good functional outcome (mRS scores of 0–3 at 3 months) in patients with acute ischaemic stroke presenting within 6 h (BASICS), within 12 h (ATTENTION), within 6–24 h (BAOCHE), or 24 h (RESCUE Japan Registry 2, ATTENTION registry) from the time last known well, treated with endovascular treatment plus best medical treatment (BMT) versus BMT alone (*p*-value for interaction 0.0004, Cochran’s Q-test for interaction testing). Only a minor proportion of patients randomised to ATTENTION and BAOCHE received IVT as part of the BMT.

**Figure 4.5.**
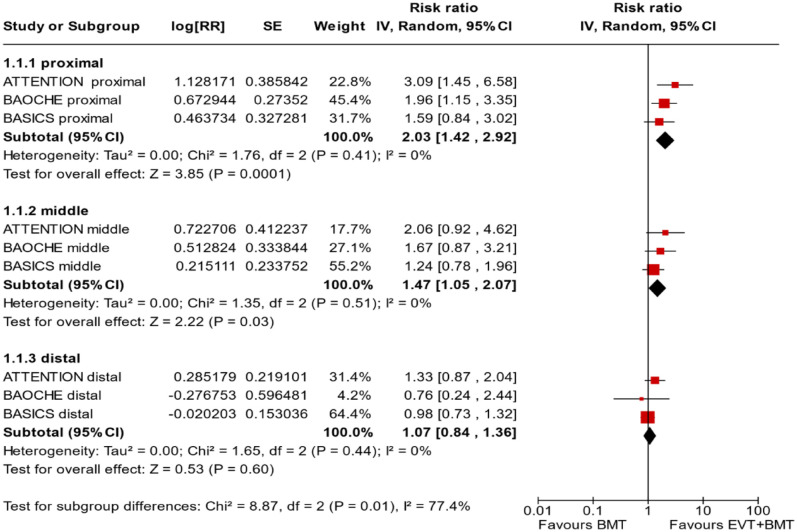
PICO 4 – Meta-analysis of randomised-controlled clinical trials (RCTs) stratified by occlusion location at baseline (*p*-value for interaction 0.01): Good functional outcome (mRS scores of 0–3 at 3 months) in patients with acute ischaemic stroke presenting within 6 h (BASICS), within 12 h (ATTENTION), or within 6–24 h (BAOCHE) from the time last known well, treated with endovascular treatment plus best medical treatment (BMT) versus BMT alone (pooled RR, random-effects meta-analysis, Cochran’s Q-test for interaction testing). Only a minor proportion of patients randomised to ATTENTION and BAOCHE received IVT as part of the BMT.

In line with the above-mentioned recent meta-analysis from RCTs using NIHSS cut-off 10,^30^ we performed a random-effects meta-analysis of randomised data stratified by the same baseline NIHSS cut-off value (Figures 4.2 and 4.3). Of note, all patients randomised into the ATTENTION trial had baseline NIHSS ⩾ 10, whereas the BEST trial (median NIHSS of randomised patients of 32 and 26 for EVT + BMT vs. BMT arms, respectively) did not provide results for this NIHSS cut-off. This analysis demonstrated a differential effect of reperfusion therapy stratified by NIHSS cutoff 10 (*p* = 0.03 for interaction). Similar interactions were detected also in non-randomised registry studies: RESCUE JAPAN LIMIT (*p* = 0.01) and ATTENTION (*p* = 0.02). For the purpose of visual demonstration, we created forest plots showing differential effect of reperfusion therapy stratified by NIHSS cutoff 10 including both randomised and non-randomised data (Figure 4.4). Because clinical severity in patients with BAO is strongly related to the location of the occlusion, we also analysed whether there is a differential effect between EVT and BMT as stratified by occlusion location (proximal, middle, distal) (Figure 4.5).

Table 3 provides details regarding the assessment of the quality of evidence for mRS score of 0–3 at 3 months in PICO 4.

**Table 3.** GRADE evidence profile for PICO 4.

Certainty assessment							No. of patients		Effect		Certainty	Importance
No of studies	Study design	Risk of bias	Inconsistency	Indirectness	Imprecision	Other considerations	Selection of reperfusion therapy (MT or IVT) candidates based on a specific clinical presentation (e.g. NIHSS cutoff – or coma on admission)	Patient selection irrespective of clinical presentation	Relative (95% CI)	Absolute (95% CI)		
mRS 0–3 RCT and NIHSS 0–9												
2	Randomised trials	Serious^a^	Not serious	Serious^b^	Serious^c^	None	26/37 (70.3%)	30/41 (73.2%)	RR 0.67 (0.23–1.99)	241 fewer per 1000 (from 563 fewer to 724 more)	⨁○○○	Critical
											Very low*	
mRS 0–3 RCT and NIHSS 10												
3	Randomised trials	Serious^a^	Not serious	Serious^b^	Serious^c^	None	197/453 (43.5%)	77/326 (23.6%)	RR 2.44 (1.77–3.36)	340 more per 1000 (from 182 more to 557 more)	⨁○○○	CRITICAL
											Very low*	
mRS 0–3, NRSI and NIHSS 0–9												
2	Non-randomised studies	Serious^d^	Not serious	Serious^b^	Not serious	None			RR 0.96 (0.78–1.19)	1 fewer per 1000 (from 1 fewer to 1 fewer)	⨁○○○	Critical
											Very low	
mRS 0–3, NRSI and NIHSS 10												
2	Non-randomised studies	Serious^d^	Not serious	Serious^b^	Not serious	None			RR 2.19 (0.84–5.76)	2 fewer per 1000 (from 6 fewer to 1 fewer)	⨁○○○	Critical
											Very low	
sICH RCT and NIHSS 0–9												
2	Randomised trials	Serious^a^	Not serious	Serious^b^	serious^c^	None	3/37 (8.1%)	0/41 (0.0%)	RR 6.78 (0.37–125.95)	0 fewer per 1000 (from 0 fewer to 0 fewer)	⨁○○○	Important
											Very low	

CI: confidence interval; RR: risk ratio; RCT: randomised controlled trials; NRSI: non-randomised studies of intervention.

a Risk of bias was assessed as serious due to high risk of bias detected in all RCTs.

b No study specifically tested efficacy in high versus low NIHSS scores.

c Serious imprecision due to low optimal information size. The total number of patients included is less than the number of patients generated by a conventional size sample calculation for a single adequately powered clinical trial.

d Risk of bias was assessed as serious using ROBINS-I tool.

* *p* < 0.03 for interaction between NIHSS 0–9 versus NIHSS ⩾ 10. *p* = 0.01 for interaction among proximal, middle, and distal locations.

### Additional information

**Figure 4.6.**
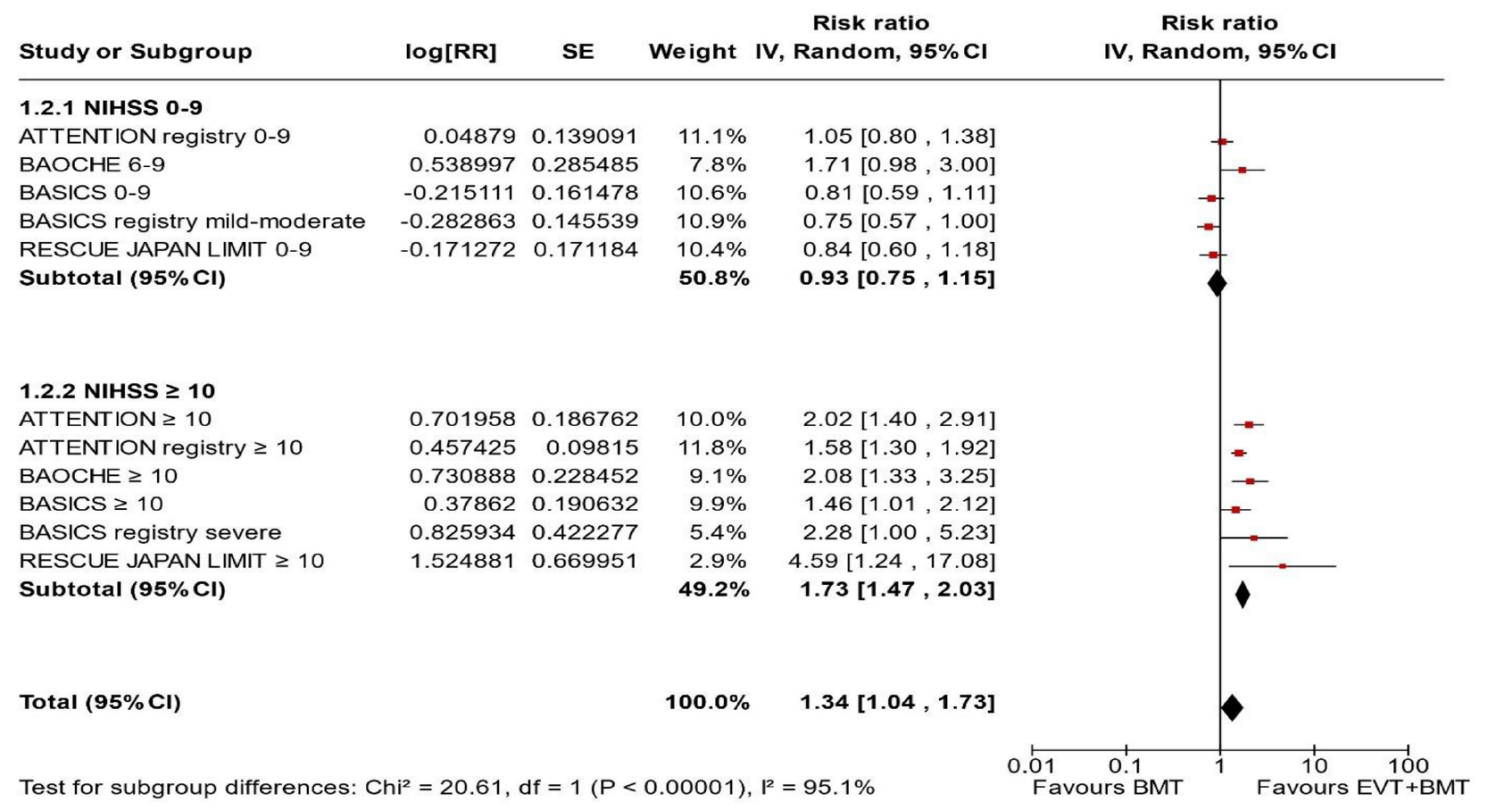
PICO 4 – Forest plot showing differential effect of reperfusion therapy stratified by NIHSS cutoff 10, including data from randomised-controlled clinical trials (RCTs) and registry studies. Good functional outcome (mRS scores of 0–3 at 3 months in all except BASICS prospective registry, where it was assessed at 1 month) in patients with acute ischaemic stroke presenting within 6 h (BASICS), within 12 h (ATTENTION), within 6–24 h (BAOCHE), or 24 h (RESCUE Japan Registry 2), or no time limit (BASICS prospective registry) from the time last known well, treated with endovascular treatment plus best medical treatment (BMT) versus BMT alone (*p*-value for interaction <0.00001, Cochran’s Q-test for interaction testing). Only a minor proportion of patients randomised to ATTENTION and BAOCHE received IVT as part of the BMT.

Schonewille et al.^2^ reported data from a prospective BAO registry stratified by stroke severity on admission (mild-to-moderate vs. severe). Severe symptoms were described as coma, locked-in state, or tetraplegia, whereas all other symptoms were considered mild-to-moderate. The registry had three arms (antithrombotics, primary IVT, and IAT. The IAT group comprised intra-arterial thrombolysis, mechanical thrombectomy, stenting, or a combination of these approaches. The outcome was assessed only at 1 month and not at 3 months as in all other studies. In addition, another major difference compared to other studies is that the primary IVT group included also subsequent IAT. For these two reasons, we only considered IAT versus no IAT (conventional, antithrombotics) comparison. For the purpose of these guidelines, we considered that ‘mild-to-moderate’ stroke severity corresponded to patients with an NIHSS < 10, whereas ‘severe’ symptoms corresponded to patients with NIHSS ⩾ 10. We created forest plots showing differential effect of reperfusion therapy stratified by NIHSS cutoff 10 including both randomised and non-randomised data (Figure 4.6). The *p*-value for interaction was <0.00001.

**Figure 4.7.**
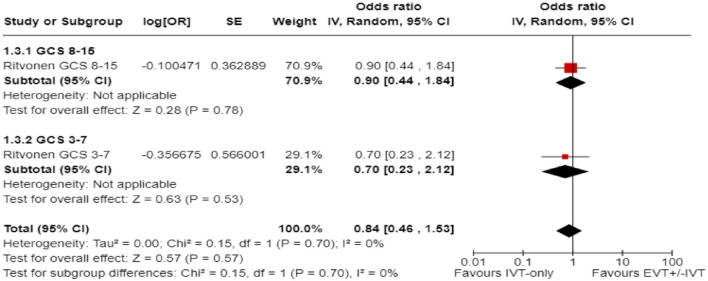
PICO 4 – Interaction testing for treatment effect between EVT ± IVT and no EVT (100% IVT) in patients with GCS 3–7 and 8–15.

Ritvonen et al.^31^ reported similar frequencies of outcomes based on the severity of the initial Glasgow Coma Scale (GCS): the 3-month mRS 0–3 in comatose (GCS < 8) and non-comatose (GCS 8–15) patients treated with EVT (±IVT) versus BMT (100% IVT) was 16.7% versus 22.2%, respectively, and the *p*-value for interaction was 0.70 (Figure 4.7).

A very large US study analysed data from the National Inpatient Sample (2018–2020), which included 5795 patients with less than 10 NIHSS points at baseline. Of those, 880 (15.4%) underwent EVT. The effect of EVT was compared to BMT. The primary outcome was discharge to home or self-care, adjusted for robust outcome predictors. A secondary analysis was performed with the same adjustments and evaluated the length of stay. After adjustments, in multivariable regression, EVT was reported to be associated with increased odds of discharge to home (OR: 1.95; (95% CI: 1.31–2.90); *p* = 0.001) and a decreased length of hospital stay (B, −0.74 (95% CI: −1.36 to −0.11); *p* = 0.02) compared with BMT. However, on 9 January 2024, an eLetter was published by the Stroke Editorial office^32^ stating that after publication, an error was discovered. Specifically, the variables for EVT and IVT were switched, and the article was retracted.

Finally, in addition to the above-mentioned interactions for the treatment effect of EVT versus no EVT stratified by baseline stroke severity, we have noticed that the direction of the forest plots comparing EVT versus BMT largely depends on the composition of the BMT group. In case it comprises mostly conventional therapy (aspirin, anticoagulation), the forest plot favoured EVT, however, when BMT was IVT in majority of the patients, there was no difference between the two arms.

### IVT versus no IVT depending on initial stroke severity

We did not identify any RCTs or subgroup data within such studies addressing the relationship between initial stroke severity and the effect of IVT on outcomes at 3 months in BAO-patients. However, given the effectiveness of IVT regardless of initial stroke severity shown in RCTs on IVT in disabling stroke,^18^ as well as evidence of its benefit in both the anterior and posterior circulation,^33,34^ it is highly likely that IVT has a beneficial effect on patients with BAO, regardless of their initial stroke severity. This is further supported by the findings of Ritvonen et al.,^31^ where no significant difference was found between IVT alone and EVT ± IVT in patients stratified by a GCS score of 8 (Figure 4.7).


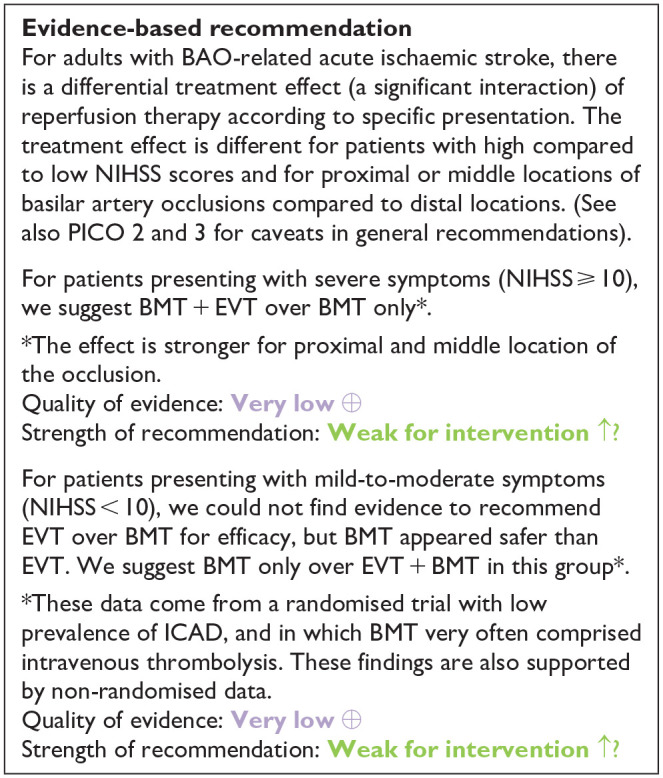


### PICO 5

For adults with BAO-related acute ischaemic stroke, does selection of reperfusion therapy (IVT and/or EVT) candidates based on a particular pc-ASPECTS compared with no specific threshold improve identification of patients with a therapy effect on outcomes?

#### Imaging of acute tissue ischemia in BAO

The extent of ischemia in BAO is most typically described by early ischaemic changes (EIC) on neuroimaging using the pc-ASPECTS score. This score was originally based on CT-angiography source images^35^ but is also applicable to non-contrast CT or MRI-based DWI imaging.^36^ Lower pc-ASPECTS scores indicate more extensive EIC. Interpretation of pc-ASPECTS on CT can be difficult due to beam hardening artifacts at the level of the temporal bones/skull base. Other less commonly used scores include the Pons-Midbrain Index (PMI) on non-contrast-CT,^37^ Pons-Midbrain and Thalamus (PMT) score on DWI-MRI,^38^ and the Critical Area Perfusion Score (CAPS) on CT-perfusion.^39^ These studies indicate that the extent of ischaemic changes seen on acute neuroimaging remains a strong prognostic factor even after successful reperfusion with EVT.

#### Analysis of current evidence

This PICO question focuses on the treatment effect of acute recanalisation therapy in patients with high versus low pc-ASPECTS points. Patients with low scores may have less or no viable tissue that could benefit from such therapy. PICO questions 2–4 describe the evidence of the effect of recanalisation treatments for BAO based on time and stroke severity. For the current PICO question, we investigated whether there is an interaction between reperfusion treatment effects in patients with high versus low pc-ASPECTS in RCTs.

While there are no randomised data regarding solely the effect of IVT based on pc-ASPECTS, but the literature search identified three potentially relevant RCTs (EVT plus BMT vs. BMT) that have already been described in detail in PICO questions 2 and 3. The subgroup (interaction) analyses in these three trials used different cut-off of pc-ASPECTS, being 9 in the BAOCHE^9^ and 8 in the BASICS^7^ and ATTENTION^8^ trials (all showing no difference). Very importantly, the median pc-ASPECTS scores of the randomised patients were rather high. In the BASICS trial, only 17% of the patients had pc-ASPECTS lower than 8 at baseline, whereas median pc-ASPECTS at 24 h based on angiography source imaging was 9 (8–10) in the EVT + BMT group and 9 (7–10) in the BMT group. Similarly, in the ATTENTION trial, only 20% of the patients had pc-ASPECTS lower than 8 at baseline (median 9 (8–10) in the EVT + BMT group and 10 (8–10) in the BMT group). In the BAOCHE trial, patients had baseline pc-ASPECTS median of 8 (7–10) in both arms.

Hence, the proportion of patients with low pc-ASPECTS scores was insufficient to perform a formal meta-analysis and draw conclusions about the interaction of the treatment effect in patients with high vs. low pc-ASPECTS. Furthermore, for two of three critical outcomes (mRS 0–2 and mortality at 3 months) data from only 1 trial (BAOCHE) were available, and for mRS 0–3 only from two trials (BASICS and ATTENTION).

### Additional information

Numerous studies have shown a strong association between poor outcomes and lower pc-ASPECTS in BAO patients, regardless of recanalisation treatment.^24,35,40–44^ In one of these studies, patients receiving recanalisation therapy (IVT or EVT) had 1-year mortality of 38% in those with pc-ASPECTS 8–10, whereas it was 66% for pc-ASPECTS < 8. In another study, patients receiving recanalisation therapy (IVT or EVT), 3-month mortality was 31% in those with pc-ASPECTS 8–10, whereas it was 64% for pc-ASPECTS < 8. In the same study, mRS 4–6 was observed in 46% and 88%, respectively. A very recent Korean study suggested some potential benefit of EVT in patients with low pc-ASPECTS^45^ based on the inverse probability of treatment weighting model for mRS score of 0–3 (33% vs. 24%, *p* = 0.03), but not based on propensity-score matching for the same outcome. For mRS score of 0–2, no difference was observed in any of the models.


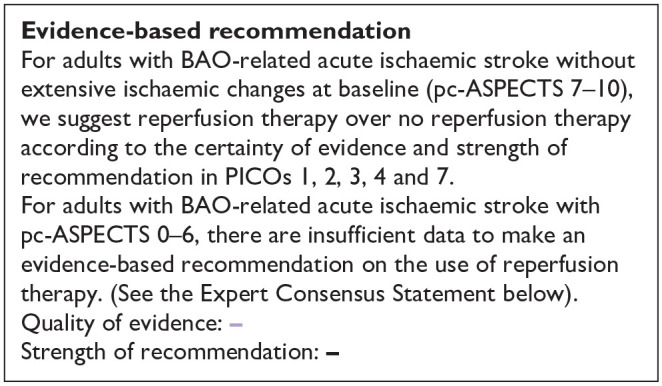



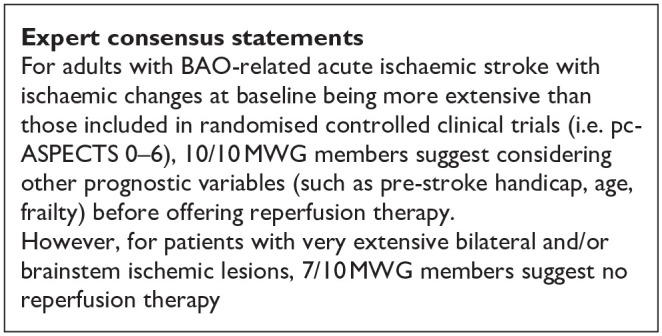


### PICO 6

For adults with BAO-related acute ischaemic stroke, does selection of reperfusion therapy (EVT or IVT) candidates based on advanced imaging criteria (perfusion, core, or collateral imaging) compared with no advanced imaging improve identification of patients with a therapy effect on outcomes?

### Analysis of current evidence

The literature search did not identify any published RCTs addressing this PICO question, but identified one post hoc analysis^46^ derived from a Chinese registry-based observational study.

The BASILAR registry has been described in PICO questions 2 and 3. Patients with evaluated Basilar Artery on Computed Tomography Angiography (BATMAN) score were included in the analysis (*n* = 828).^46^ The primary efficacy outcome was good functional status, defined as mRS scores of 0–3 at 3 months. The secondary efficacy outcomes included functional independence defined as mRS score of 0–2 at 3 months, and successful reperfusion.

In all three categories of the BATMAN score (0–3, 4–6, and 7–10), EVT + BMT was associated with higher odds in achieving better outcomes and lower mortality compared to BMT (approx. 80% conventional treatment with antiaggregatory or anticoagulation). *p*-value for interaction was 0.52.

**Figure 5.1.**
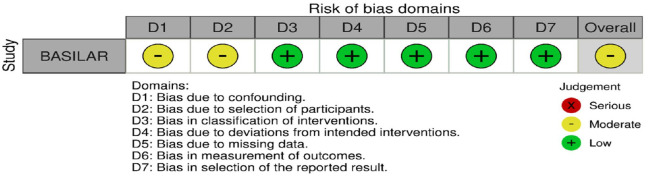
PICO 6 – Risk of bias in an observational study.

The study presented moderate confounding bias (Figure 5.1), since there were several significant baseline differences between the different treatment groups.

Thus, the only study relevant to this PICO question evaluated the effect of collateral flow. No other advanced imaging criteria were found to be tested.


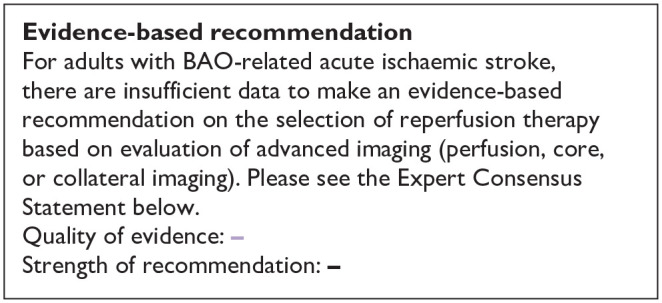



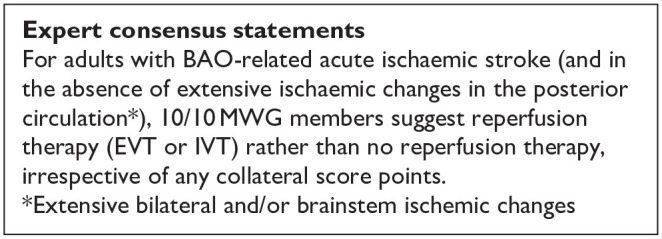


### PICO 7

For adults with BAO-related AIS without contraindication for IVT, does direct EVT compared to EVT plus IVT improve outcomes?

### Analysis of current evidence

The literature search identified no RTs and three prospective cohort studies as relevant for this PICO.

Nie et al.^47^ reported post EVT outcomes in patients with and without concurrent IVT in a prospective multicentre RESCUE-RE cohort study accompanied by a meta-analysis of the existing literature. The RESCUE-RE study enrolled patients with AIS due to vertebrobasilar occlusion that were 18 years or older, had a pre-stroke mRS score of 0–2 and were followed up for 3 months. IVT, if indicated, was administered within 4.5 h from symptom onset (0.9 mg alteplase/kg). Between July 2018 to October 2020, 1701 patients were enrolled in the registry, of which 321 patients were included in the study.

Singer et al.^48^ reported post-EVT outcomes in a retrospective multicentre cohort study, ENDOSTROKE. This study enrolled both prospectively and retrospectively patients with any large vessel occlusion in the anterior or posterior circulation, who were 18 years of age or older and in whom EVT was attempted. The study included a subgroup of 148 patients with attempted EVT for BAO in whom 3-months follow up data were available. Concurrent IVT was permitted in their study (not stated to how many it was administered), however, patients experiencing thrombolysis-related recanalisation prior to EVT were excluded. The primary outcome was mRS score of 0–2 at 3 months. The main angiographic outcome was recanalisation defined as a final TICI score of 2b or greater.

Siow et al.^49^ reported results from a retrospective multicentre cohort study. Patients were included if they underwent EVT for acute BAO and had a pre-stroke mRS score of 0–2. Between January 2015 and December 2019, 322 patients who met the inclusion criteria were included in the study. Patients received IVT (0.9 mg/kg alteplase) if they had no contraindications and could be treated within 4.5 h of symptom onset. The primary outcome was mRS score of 0–3 at 3 months.

Nappini et al.^50^ reported results of a secondary analysis from a national prospective registry of EVT. Patients were included if they underwent EVT for BAO, either with or without IVT with tissue plasminogen activator (time window of 4.5 h from symptom onset). The outcomes were recanalisation status, and different dichotomizations of the 90-day mRS. Between 2011 and 2017, 464 who underwent EVT for BAO were included in the registry. Overall, patients treated with EVT alone had less favourable baseline characteristics, including higher NIHSS and higher prevalence of baseline co-morbidities and anticoagulant treatment. Clinical outcomes were better in patients receiving bridging IVT in the unadjusted analysis, but this did not hold true after adjusting for confounding variables. In a post-hoc subgroup analysis in patients treated with EVT within 6 hours from symptom onset, patients receiving bridging IVT had reduced risk of death and a shift towards better 90-day mRS in the adjusted analysis.

Singh Kohli et al.^51^ report a small single-centre series of 31 BAO patients undergoing EVT, 22 of which underwent direct EVT, while 9 received bridging IVT. Baseline characteristics and time to treatment were generally more favourable in the patients who received bridging IVT (time window of 4.5 h from symptom onset). Unadjusted clinical and technical outcomes were more favourable in the bridging IVT group; however, the small group size did not permit adjusted analysis.

**Figure 6.1.**
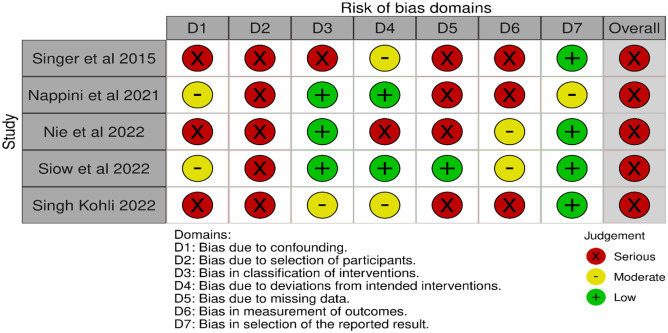
PICO 7 – Risk of bias for the non-randomised trials included in PICO 7.

Risk of bias assessment for the included non-randomised studies (Figure 6.1) showed serious risk of bias for all included studies.

**Figure 6.2.**
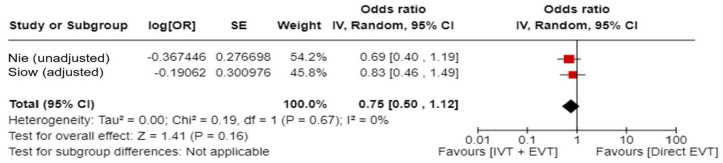
PICO 7 – Meta-analysis of observational studies: Good functional outcome (mRS scores 0–3 at 90 days) in adults with acute ischaemic stroke due to BAO, treated with direct endovascular thrombectomy versus intravenous thrombolysis and endovascular thrombectomy (pooled OR, random-effects meta-analysis).

We conducted several meta-analyses to provide a quantitative synthesis of the results (Figures 6.2–6.6), and we state in the figure if the available estimates were adjusted for potential confounders. Briefly, point estimates of critical outcomes (all mRS-related outcomes) were in favour of combined IVT and EVT treatment. Statistically significant differences were found for shift mRS and adjusted mRS score of 0–2 at 3 months. For sICH and mTICI, no difference was found. For mortality at 90 days, only data from one study were available, hence, no meta-analysis was conducted. The adjusted ORs for this outcome with combined treatment compared to direct EVT was 1.79 (0.87–3.70).

**Figure 6.3.**
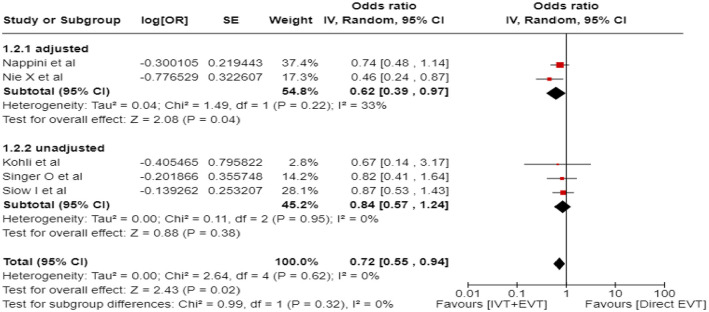
PICO 7 – Meta-analysis of observational studies: Good functional outcome (mRS scores of 0–2 at 3 months) in adults with acute ischaemic stroke due to BAO, treated with direct endovascular thrombectomy vs. intravenous thrombolysis and endovascular thrombectomy (pooled OR, random-effects meta-analysis).

**Figure 6.4.**
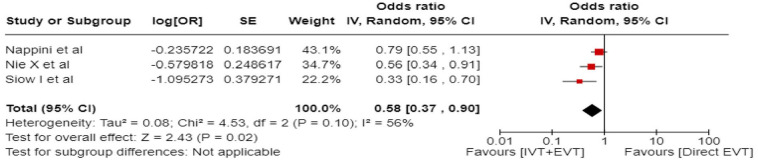
PICO 7 – Meta-analysis of observational studies: Good functional outcome (shift mRS scores of at 3 months) in adults with acute ischaemic stroke due to BAO, treated with direct endovascular thrombectomy versus intravenous thrombolysis and endovascular thrombectomy (pooled adjusted OR, random-effects meta-analysis).

**Figure 6.5.**
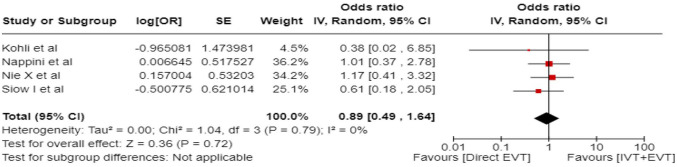
PICO 7 – Meta-analysis of observational studies: Symptomatic intracranial haemorrhage post treatment in adults with acute ischaemic stroke due to BAO, treated with direct endovascular thrombectomy versus intravenous thrombolysis and endovascular thrombectomy (pooled adjusted OR, random-effects meta-analysis).

**Figure 6.6.**
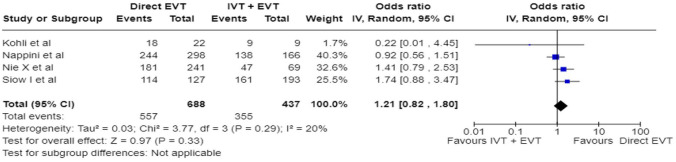
PICO 7 – Meta-analysis of observational studies: Favourable recanalisation (mTICI 2b/3 post treatment) in adults with acute ischaemic stroke due to BAO, treated with intravenous thrombolysis and endovascular thrombectomy versus direct endovascular thrombectomy (pooled adjusted OR, random-effects meta-analysis).

Table 4 provides details regarding the assessment of the quality of evidence in PICO 7.

**Table 4.** GRADE evidence profile for PICO 7.

Certainty assessment							No. of patients		Effect		Certainty	Importance
No. of studies	Study design	Risk of bias	Inconsistency	Indirectness	Imprecision	Other considerations	direct EVT	IVT + EVT	Relative (95% CI)	Absolute (95% CI)		
mRS 0–3 at 90 days observational												
2	Non-randomised studies	Serious^a^	Not serious	Not serious	Serious^b^	None	154/436 (35.3%)	81/196 (41.3%)	OR 0.75 (0.50–1.12)	68 fewer per 1000 (from 153 fewer to 28 more)	⨁⨁○○	Critical
											Low	
mRS 0–2 at 90 days observational												
5	Non-randomised studies	Serious^c^	Not serious	Not serious	Serious^b^	None	272/682 (39.9%)	161/360 (44.7%)	OR 0.70 (0.48–1.05)	86 fewer per 1000 (from 168 fewer to 12 more)	⨁⨁○○	Critical
											Low	
shift mRS 90 days observational												
3	Non-randomised studies	Serious^a^	Not serious	Not serious	Serious^b^	None			OR 0.58 (0.37–0.90)	1 fewer per 1000 (from 1 fewer to 0 fewer)	⨁⨁○○	Critical
											Low	
TICI 2B/3 90 days observational												
4	Non-randomised studies	Serious^a^	Not serious	Not serious	Serious^b^	None	604/754 (80.1%)	308/371 (83.0%)	OR 0.89 (0.40–1.96)	17 fewer per 1000 (from 169 fewer to 75 more)	⨁⨁○○	Critical
											Low	
sICH observational												
4	Non-randomised studies	Serious^a^	Not serious	Not serious	Serious^b^	None	41/741 (5.5%)	18/363 (5.0%)	OR 1.20 (0.35–4.07)	9 more per 1000 (from 32 fewer to 126 more)	⨁⨁○○	Critical
											Low	

CI: confidence interval; OR: odds ratio.

a Serious risk of bias due to serious confounding reported in both studies implemented for this outcome according to ROBINS-I tool for observational studies.

b Serious imprecision due to low optimal information size. The total number of patients included is less than the number of patients generated by a conventional size sample calculation for a single adequately powered clinical trial.

c Serious risk of bias due to serious confounding reported in studies implemented for this outcome according to ROBINS-I tool for observational studies.

### Additional information

In the anterior circulation, non-inferiority of direct EVT could not be proven in a patient-level meta-analysis of all anterior circulation randomised direct-to-EVT trials.^52^ Of note, an RCT of tenecteplase prior to EVT compared to EVT alone is ongoing in patients with BAO (POST-ETERNAL).


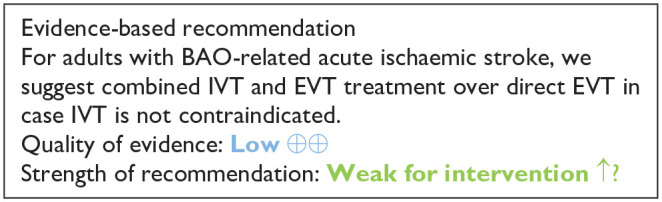


### PICO 8

For adults with BAO-related acute ischaemic stroke, does mechanical thrombectomy using direct aspiration as the first-line strategy compared with a stent retriever as the first-line strategy improve outcomes?

### Analysis of current evidence

Stent retriever thrombectomy was the preferred technique in pivotal trials demonstrating benefits of mechanical thrombectomy plus BMT over BMT alone in the acute anterior circulation strokes.^4^ Based on the expert opinion in the latest ESO-ESMINT guideline for Mechanical Thrombectomy in Acute Ischaemic Stroke.^5^ A Direct Aspiration First Pass Technique (ADAPT) may be used as a standard first-line treatment, followed by stent retriever thrombectomy as a rescue therapy if needed.

The literature search did not identify any completed RCTs comparing the different first-line treatment techniques in patients with BAO. For the comparison of the first-line contact aspiration and stent-retriever thrombectomy, the literature search identified one post hoc analysis of an RCT,^53^ seven registry-based observational studies,^54–60^ and four single-centre retrospective observational studies.^61–64^

In the post hoc analysis of the BASICS trial by Knapen et al.,^53^ 127 patients with BAO who underwent EVT with either direct aspiration (*n* = 60) or stent retriever thrombectomy (*n* = 67) as the first-line approach were included. The primary outcome was mRS score of 0–3 at 3 months. Secondary outcomes included mRS score at 3 months, procedure duration, mortality at 3 months, and sICH.

The retrospective analysis of two stroke registries by Abdelrady et al.^57^ investigated the influence of the frontline endovascular technique in 128 patients with BAO between January 2015 and December 2019. Of those 128, 33 were treated with contact aspiration, 35 with stent-retriever thrombectomy, 35 underwent combined technique (contact aspiration + stent-retriever), and in 25 patients the technique was switched. The outcomes included first pass mTICI 3 reperfusion, mTICI 2b-3, and mTICI 2c-3, as well as favourable clinical outcome (mRS score 0–2 at 3 months). The authors also reported frequency of sICH.

The STAR registry^58^ was a prospective, multicentre registry in the United States and Germany, recruiting patients between June 2014 to December 2018. Of 3045 patients, 345 presenting with posterior circulation stroke and treated with mechanical thrombectomy using modern devices were included in the analysis comparing different techniques (contact aspiration, stent-retriever, combined approach). Of the 345 patients, 121 were treated with contact aspirations, 90 patients with stent-retriever thrombectomy, and the rest with combined approach. The outcome measures included successful recanalisation mTICI 2b-3, clinical outcome (mRS score 0–2 at 3 months) and frequency of sICH.

In the study by Baik et al.,^59^ 161 patients from two university hospital stroke registries with acute BAO referred for mechanical thrombectomy between March 2013 and December 2019 were enrolled, out of which 43 underwent contact aspiration and 118 stent-retriever thrombectomy. The authors reported mTICI 2b-3, mTICI 3, clinical outcome mRS score of 0–2 at 3 months, mortality at 3 months, and frequency of sICH, all outcomes stratified according to the angiographic characteristics of the occlusion.

The MR CLEAN Registry^60^ was a nationwide prospective registry of consecutive patients who underwent EVT in the Netherlands between March 2014 and December 2018. Two hundred five patients with intracranial proximal occlusion in the posterior circulation (basilar artery, intracranial part of the vertebral artery and posterior cerebral artery), who underwent EVT with contact aspiration (*n* = 71) or stent retriever thrombectomy (*n* = 134) as the first-line approach were analysed. Outcome measures included mRS score (0–2 and 0–3 at 3 months) and final eTICI reperfusion grade. Mortality and frequency of sICH was also reported.

A post-hoc analysis from the ETIS (Endovascular Treatment in Ischaemic Stroke) registry by Gory et al.^54^ included 100 patients presenting with BAO between March 2010 and October 2016 at 3 comprehensive stroke centres. Forty-six patients underwent first-line contact aspiration and 54 first-line stent-retriever thrombectomy. The reported outcome measures included mTICI 2b-3, mTICI 3, mRS score of 0–2 at 3 months, 3-month mortality, and sICH.

The Tama-REgistry of Acute Thrombectomy (TREAT) was a regionwide, multicentre, retrospective observational registry in Japan. The post hoc analysis by Kaneko et al.^55^ comprised of 48 patients with acute BAO who underwent EVT between January 2015 and December 2017, out of which 12 patients underwent first-line contact aspiration and 33 first-line stent-retriever thrombectomy. The primary outcomes were functional outcomes (mRS scores of 0–2 and 0–3) and all-cause mortality at 3 months.

The ENTHUSE (Endovascular thrombectomy for acute basilar artery occlusion) was retrospective, multicentre, observational study, conducted at three high-volume stroke centres in South Korea.^56^ The post hoc analysis comprised of 212 patients with acute BAO who underwent EVT between January 2011 and August 2017, out of which 67 underwent first line contact aspiration and 145 first-line stent-retriever thrombectomy. The reported outcome measures included mTICI 2b-3, mTICI 3, mRS score 0–2 at 3 months, and 3-month mortality.

A single centre retrospective study by Choi et al.^63^ included 50 patients with acute BAO treated with contact aspiration (*n* = 34) or stent-retriever thrombectomy (*n* = 16) between March 2016 to December 2019. The reported outcome measures included successful reperfusion mTICI 2b-3, mRS score of 0–2 at 3 months, 3-month mortality, and sICH.

A single-centre retrospective study by Lee et al.^62^ included 38 patients with 40 vertebrobasilar occlusions, that were treated with contact aspiration (*n* = 11) or stent-retriever thrombectomy (*n* = 29) between March 2010 to December 2017. The reported outcome measures included mTICI 2b-3 and mRS score of 0–2 at 3 months.

A single-centre study by Sangpetngam et al.^64^ retrospectively analysed 66 patients with vertebrobasilar occlusions treated with EVT (the authors reported 9 patients with vertebral artery occlusion among 61 patients with successful reperfusion). Thirty-two patients were treated with first-line contact aspiration and 34 patients with first-line stent-retriever thrombectomy. The reported outcomes included mTICI 2b-3, and mRS score of 0–2.

A single-centre study by Son et al.^61^ retrospectively analysed 31 patients with acute BAO treated with EVT between March 2010 to December 2013. Eighteen patients were treated with first-line contact aspiration and 13 patients with first-line stent-retriever thrombectomy. The reported outcomes included mTICI 2b-3, mTICI 3, and mRS score of 0–2.

The PC-SEARCH Thrombectomy (Posterior Circulation Ischaemic Stroke Evaluation: Analysing Radiographic and Intraprocedural Predictors for Mechanical Thrombectomy) registry^65^ was a multicentre retrospective collaboration from eight high-volume centres in the United States consisting of consecutive patients with BAO treated with EVT between January 2015 and December 2021. Out of 383 patients included in the retrospective analysis, 219 underwent first-line contact aspiration and 164 received first-line stent-retriever thrombectomy. The reported outcome measures included mTICI 2b-3, mRS scores of 0–2 and 0–3 at 3 months, and rate of sICH.

**Figure 7.1.**
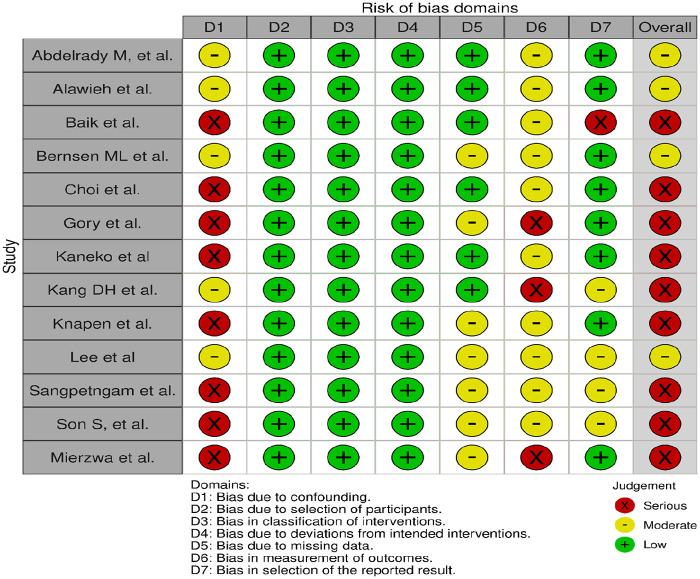
PICO 8 – Risk of bias of the studies.

Risk of bias for the included studies is presented in Figure 7.1.

**Figure 7.2.**
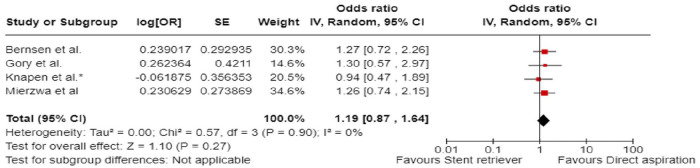
PICO 8 – Meta-analysis of observational studies (except for *post hoc analysis of the BASICS RCT): Good functional outcome (mRS scores of 0–3 at 3 months) in adults with acute ischaemic stroke due to acute BAO, treated with EVT using direct aspiration versus stent retriever as the first-line strategy (pooled OR, random-effects meta-analysis).

We performed several random-effects meta-analyses comparing the two techniques of interest (Figures 7.2–7.6).

**Figure 7.3.**
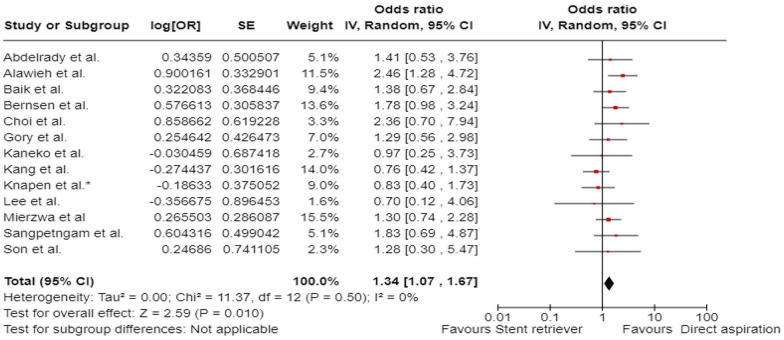
PICO 8 – Meta-analysis of observational studies (except for *post hoc analysis of the BASICS RCT): Favourable functional outcome (mRS scores of 0–2 at 3 months) in adults with acute ischaemic stroke due to acute BAO, treated with EVT using direct aspiration versus stent retriever as the first-line strategy (pooled OR, random-effects meta-analysis).

**Figure 7.4.**
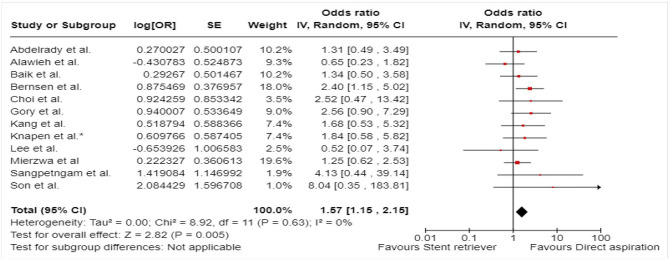
PICO 8 – Meta-analysis of observational studies (except for *post hoc analysis of the BASICS RCT): Successful recanalisation (mTICI 2B-3) in adults with acute ischaemic stroke due to acute BAO, treated with EVT using direct aspiration versus stent retriever as the first-line strategy (pooled OR, random-effects meta-analysis).

**Figure 7.5.**
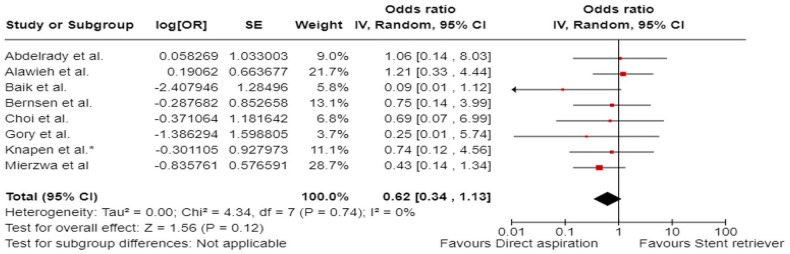
PICO 8 – Meta-analysis of observational studies (except for *post hoc analysis of the BASICS RCT): Symptomatic ICH in adults with acute ischaemic stroke due to acute BAO, treated with EVT using direct aspiration versus stent retriever as the first-line strategy (pooled OR, random-effects meta-analysis).

**Figure 7.6.**
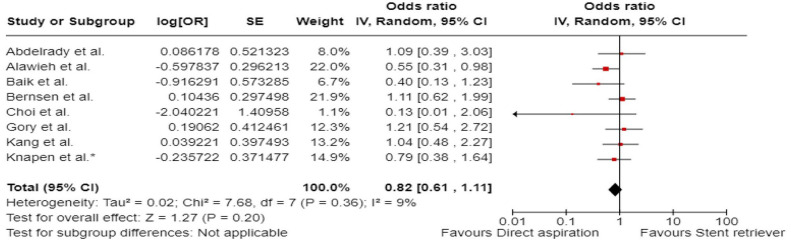
PICO 8 – Meta-analysis of observational studies (except for *post hoc analysis of the BASICS RCT): Mortality at 90 days in adults with acute ischaemic stroke due to acute BAO, treated with EVT using direct aspiration versus stent retriever as the first-line strategy (pooled OR, random-effects meta-analysis).

**Figure 7.7.**
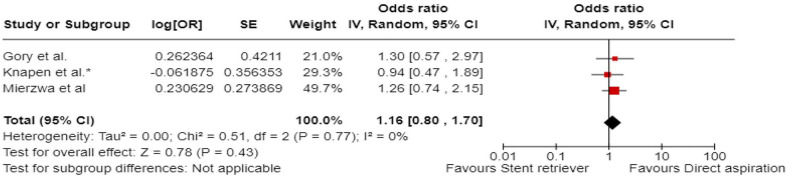
PICO 8 – Sensitivity meta-analysis of observational studies (except for *post hoc analysis of the BASICS RCT): Good functional outcome (mRS scores of 0–3 at 3 months) in adults with acute ischaemic stroke due to acute BAO, treated with EVT using direct aspiration vs. stent retriever as the first-line strategy (pooled OR, random-effects meta-analysis).

Sensitivity analyses (after excluding studies comprising all posterior-circulation strokes) of critical and important outcomes are depicted in Figures 7.7–7.11.

**Figure 7.8.**
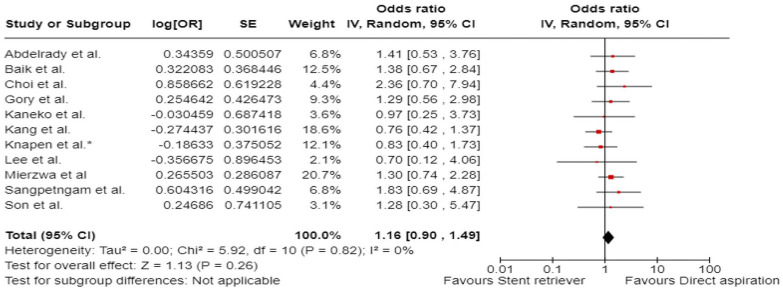
PICO 8 – Sensitivity meta-analysis of observational studies (except for *post hoc analysis of the BASICS RCT): Favourable functional outcome (mRS scores of 0–2 at 3 months) in adults with acute ischaemic stroke due to acute BAO, treated with EVT using direct aspiration vs. stent retriever as the first-line strategy (pooled OR, random-effects meta-analysis).

**Figure 7.9.**
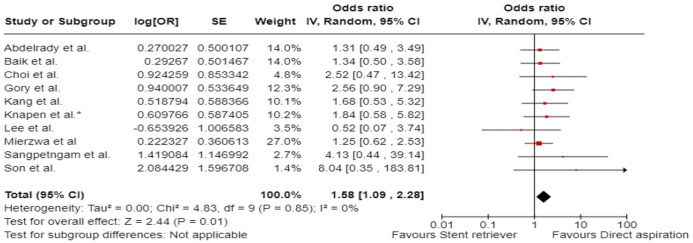
PICO 8 – Sensitivity meta-analysis of observational studies (except for *post hoc analysis of the BASICS RCT): Successful recanalisation (mTICI 2B-3) in adults with acute ischaemic stroke due to acute BAO, treated with EVT using direct aspiration versus stent retriever as the first-line strategy (pooled OR, random-effects meta-analysis).

**Figure 7.10.**
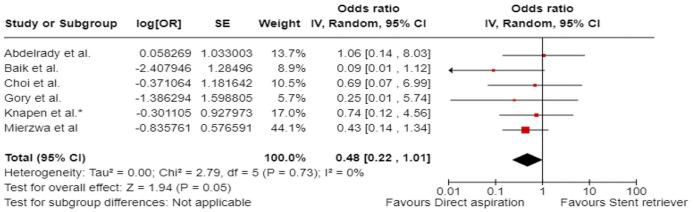
PICO 8 – Sensitivity meta-analysis of observational studies (except for *post hoc analysis of the BASICS RCT): Symptomatic intracranial haemorrhage in adults with acute ischaemic stroke due to acute BAO, treated with EVT using direct aspiration versus stent retriever as the first-line strategy (pooled OR, random-effects meta-analysis).

**Figure 7.11.**
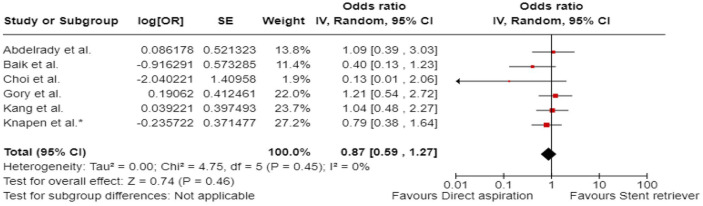
PICO 8 – Sensitivity meta-analysis of observational studies (except for *post hoc analysis of the BASICS RCT): Mortality at 90 days in adults with acute ischaemic stroke due to acute BAO, treated with EVT using direct aspiration vs. stent retriever as the first-line strategy (pooled OR, random-effects meta-analysis).

Table 5 provides details regarding the assessment of the quality of evidence for critical and important outcomes evaluated in PICO 8.

**Table 5.** GRADE evidence profile for PICO 8.

Certainty assessment							No. of patients		Effect		Certainty	Importance
No. of studies	Study design	Risk of bias	Inconsistency	Indirectness	Imprecision	Other considerations	EVT using direct aspiration as the first-line strategy	stent retriever as the first-line strategy	Relative (95% CI)	Absolute (95% CI)		
Successful recanalisation (TICI 2b-3); TICI: thrombolysis in cerebral ischemia: observational studies												
12	Non-randomised studies	Serious^a^	Not serious	Not serious	Not serious	None	527/602 (87.5%)	731/899 (81.3%)	OR 1.57 (1.15–2.15)	59 more per 1000 (from 20 more to 90 more)	⨁○○○	IMPORTANT
											Very low	
mRS 0–3 at 90 days: Observational studies												
4	Non-randomised studies	Serious^a^	Not serious	Not serious	Serious^b^	None	120/259 (46.3%)	171/404 (42.3%)	OR 1.19 (0.87–1.64)	43 more per 1000 (from 34 fewer to 123 more)	⨁○○○	CRITICAL
											Very low	
Favourable outcome (mRS 0–2) at 90 days: Observational studies												
13	Non-randomised studies	Serious^a^	Not serious	Not serious	Not serious	None	242/607 (39.9%)	314/928 (33.8%)	OR 1.34 (1.07–1.67)	68 more per 1000 (from 15 more to 122 more)	⨁⨁○○	IMPORTANT
											Low	
Symptomatic intracranial haemorrhage (sICH): Observational studies												
8	Non-randomised studies	Serious^a^	Not serious	Not serious	Serious^b^	None	17/445 (3.8%)	48/671 (7.2%)	OR 0.62 (0.34–1.13)	26 fewer per 1000 (from 46 fewer to 9 more)	⨁○○○	IMPORTANT
											Very low	
Mortality at 90 days: Observational studies												
8	Non-randomised studies	Serious^a^	Not serious	Not serious	Not serious	None	135/451 (29.9%)	204/666 (30.6%)	OR 0.82 (0.61–1.11)	40 fewer per 1000 (from 94 fewer to 23 more)	⨁○○○	IMPORTANT
											Very low	

CI: confidence interval; OR: odds ratio.

a Serious risk of bias due to serious confounding reported in some of these studies implemented for this outcome according to ROBINS-I tool for observational studies.

b Serious imprecision due to low optimal information size. The total number of patients included is less than the number of patients generated by a conventional size sample calculation for a single adequately powered clinical trial.

### Additional information

We also identified four observational studies^66–69^ that reported data on endovascular technique used in the posterior circulation stroke thrombectomy. However, the authors of the above-mentioned studies reported results for stent-retriever thrombectomy alone and combined (simultaneous) contact aspiration plus stent-retriever thrombectomy. Based on the consensus of the MWG, these studies were excluded from the meta-analysis as the combined approach was considered as a separate endovascular technique. Data from these four studies listed below favour direct aspiration as the first-line strategy.

The RELOBA (Registro Endovascolare Lombardo Occlusione Basilar Artery) study group included 102 patients with acute BAO treated endovascularly in 12 centres in the region of Lombardy (Italy) between January 2010 and December 2015.^66^ Successful reperfusion TICI 2b-3 was achieved in 20/27 (74.1%) patients treated with contact aspiration and in 47/65 (72.3%) patients with stent-retriever thrombectomy (alone or combined).

A study by Li et al.^67^ was a single-centre retrospective study of 68 patients with acute BAO who underwent EVT between January 2014 and December 2016. The primary outcome, mRS score of 0–2 at 3 months, was achieved in 5/7 (71.4%) patients treated with contact aspiration and in 20/50 (40.0%) patients treated with stent-retriever thrombectomy (including 47 patients treated with stent-retriever alone and 3 patients treated with combined technique).

A retrospective analysis of prospectively collected data by Monteiro et al.^68^ comprised of 83 patients with acute BAO between January 2013 to December 2020. Twenty-three patients were treated with contact aspiration, 20 patients with stent-retriever alone, and 40 patients with combined technique. The reported outcomes included successful reperfusion TICI 2b-3, first pass TICI 2c-3 and mRS score of 0–2.

The CICAT was a prospective registry including all stroke patients in Catalonia from January 2016 to January 2020. The post-hoc analysis by Terceno et al.^69^ included 298 patients with posterior circulation stroke (out of which 216 patients had BAO). The data on endovascular technique were available in 261/298 patients. The mRS score of 0–2 in 3 months was achieved in 27/62 (43.5%) patients treated with contact aspiration, in 32/108 (29.6%) treated with stent-retriever alone, and in 33/91 (36.3%) with a combined technique.

A study by Gerber et al.^70^ reported recanalisation according to AOL instead of mTICI. AOL 2–3 was achieved in 9/13 (69%) stent retriever patients, whereas it was 17/20 (85%) in the aspiration arm. In order to maintain consistency in the reported outcome (mTICI vs. AOL), this study was excluded from the meta-analysis for reperfusion outcomes.


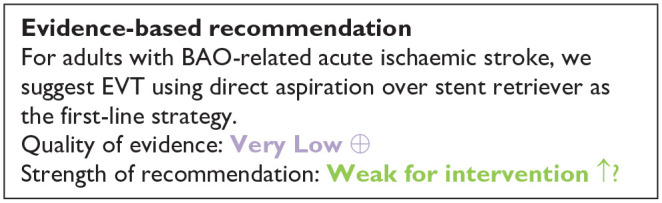


### PICO 9

For adults with BAO-related acute ischaemic stroke and with suspected intracranial atherosclerotic disease and BA stenosis, does PTA and/or stenting of the basilar artery plus EVT compared with EVT alone improve outcomes?

### Analysis of current evidence

The literature search identified no RCTs addressing this PICO question. As ICAD is often diagnosed after EVT rather than before, RCTs are unlikely to be performed. We identified one observational study conducted in China that addressed this PICO in a subgroup analysis of patients with ICAD.^71^ The proportion of mRS score of 0–3 was 33% in EVT alone (40% in successfully recanalised, 15.9% in non-recanalised), compared to 26.8% in EVT plus rescue treatment (*p* = 0.004). The 90-day mortality differed little between the groups; 46.4% in EVT alone (34.9% in successfully recanalised, 79.5% in non-recanalised), compared to 47.7% in EVT plus rescue treatment. Hence, among patients in whom EVT was not successful, those who underwent rescue PTA and/or stenting had better clinical outcomes, lower mortality, and lower sICH, although non-significant) rates than those in whom no rescue percutaneous transluminal angioplasty (PTA) and/or stenting was performed. In the EVT arms of recent BAOCHE and ATTENTION RCTs, angioplasty/stenting was performed in 39.8%–54.5%. Both trials recruited Chinese patients having a high prevalence of ICAD, and EVT alone versus EVT plus rescue treatment in ICAD patients was not addressed in either study.^8,9^ Furthermore, in a subgroup analysis of the ATTENTION trial, patients with underlying ICAD as the cause of stroke, did not show a clear benefit from EVT compared with BMT (OR: 1.59, 95% CI: 0.91–2.68).^8^

**Figure 8.1.**
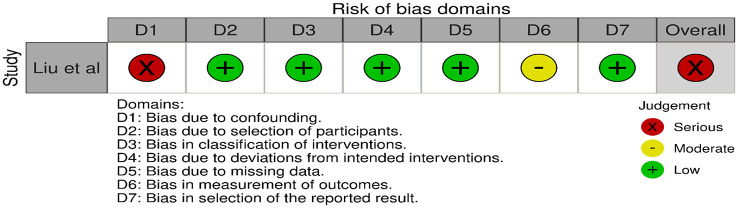
PICO 9 – Bias evaluation of the observational studies.

Bias of the aforementioned observational study is showed in Figure 8.1. No meta-analysis was performed.

### Additional information

ICAD is a disease of major intracranial arteries with different manifestations, ranging from subtle arterial wall thickening to severe stenosis with vulnerable atherosclerotic plaques.^72^ Depending on the study, the basilar artery is the most common or second most common affected intracranial vessel.^72,73^ ICAD prevalence shows marked racial/ethnical differences. In the Northern Manhattan Stroke Study, a prospective registry study of 714 patients, ICAD was the presumed cause of stroke in 9% of Caucasian patients, 15% of Hispanic, and 17% of African-American patients.^74^ ICAD is responsible for 10%–48% of all large-vessel occlusion (LVO) strokes; it is particularly common in Asia but even in Europe, up to 1 of 10 LVO strokes are caused by ICAD.^75,76^ In the Trevo endovascular registry, which included mainly European patients, ICAD accounted for only 10% of all EVT cases of BAO,^77^ while in the Chinese ATTENTION and BEST trials, atherosclerosis was the underlying stroke aetiology in 44%–56% of cases.^6,8^

Studies comparing EVT in patients with BAO due to ICAD versus other stroke mechanisms found nominally higher numbers of rescue PTA and/or stenting in patients with underlying ICAD,^77–79^ although proportions differed significantly only in one study.^80^ Despite these rescue treatments, EVT in BAO due to underlying ICAD was in most studies associated with poorer outcomes, longer procedure times and in some studies, less successful reperfusion compared to other stroke mechanisms,^77,79,81^ whereas one study found no difference in outcomes between BAO caused by ICAD compared to non-ICAD.^80^ ICAD-related occlusions are prone to re-occlude, occurring in up to 40% of patients.^82^ While the apposition thrombus that has formed adjacent to the atherosclerotic plaque can be removed by EVT alone, new thrombus may form at the thrombogenic plaque surface, thereby leading to re-occlusion This risk may be even higher after an endovascular attempt, as the traumatic fibrous cap disruption and vessel wall trauma caused by endovascular devices increase thrombogenicity even further. PTA with or without stenting can eliminate or reduce the stenosis caused by the atherosclerotic lesion, and in theory, stenting may reduce the risk of re-occlusion by covering the thrombogenic lesion. On the other hand, PTA/stenting may cause perforator occlusions by pushing plaque fragments into small perforator orifices, requiring dual antiplatelet therapy, which increases the risk of haemorrhage, particularly in cases with concomitant IVT.^83^

Two studies specifically assessed rescue therapy in failed EVT for BAO, but were not confided to patients with underlying ICAD, although ICAD patients accounted for the majority that underwent rescue treatment (77.3%–88.5%), with the comparator being all patients with successful or failed EVT in one study,^84^ and only failed EVT in the other.^85^ If we put aside successful recanalisation in non-ICAD patients after EVT alone, those who achieved recanalisation after rescue therapy had better prognosis than those not recanalised at all. Of note, compared to failed EVT without rescue therapy, the rate of sICH was lower in the EVT plus rescue therapy group in one study (14.2% compared to 4.2%, *p* = 0.002),^84^ while the other study reported small numbers of events (one case of sICH in each group) without significant difference.^85^

Another approach in case of severe underlying basilar artery stenosis after EVT is use of antithrombotic agents such as GP IIb/IIIa inhibitors. One study compared this treatment to angioplasty with or without stenting in 55 patients and found no difference in sICH, mortality, or functional outcome between the two strategies.^56^


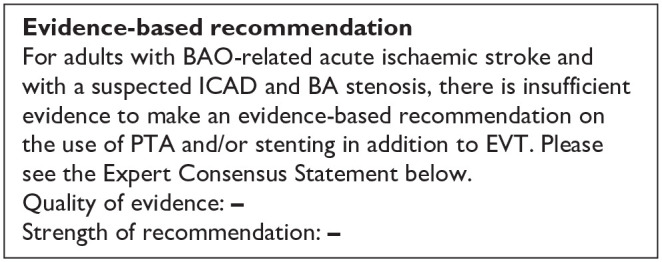



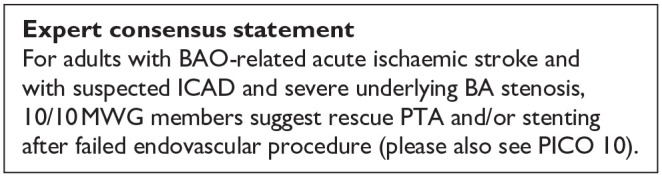


### PICO 10

For adults with BAO-related acute ischaemic stroke subjected to reperfusion therapy (EVT or IVT), does add-on antithrombotic treatment during EVT or within 24 h after IVT or EVT compared with no add-on antithrombotic treatment improve outcomes?

### Analysis of current evidence

The literature search did not identify any published RCTs addressing the PICO question, but eight non-randomised studies were identified: six observational registry-based studies,^86–91^ one non-randomised trial,^92^ and one study combining data from a prospective registry and an open-label, single-arm trial.^93^ Seven studies^86–92^ compared add-on tirofiban, whereas one study eptifibatide^93^ to no add-on antithrombotic medication for patients undergoing EVT ± IVT. Studies that included solely BΑO or dominant vertebral artery occlusion patients will be described in this section, whereas reports from studies with a subgroup of BAO patients or secondary analysis from posterior circulation studies (with uncertain proportion of BAO patients) will be presented in additional information below.

The study by Chen et al.^88^ compared patients treated with EVT for BAO based on whether they did (*n* = 363) or did not (*n* = 282) receive add-on tirofiban. IVT was administered for 17.1% and 20.2%, whereas IAT for 8.0% and 18.8% of the patients, respectively. The cohort was drawn from the Chinese, nationwide, prospective BASILAR registry comprising consecutive adult patients with BAO within 24 h of symptom onset between January 2014 and May 2019. Patients with pre-stroke mRS ⩾ 3 were excluded. Tirofiban was administered intravenously 0.4 μg/kg/min for 30 min followed by 0.1 μg/kg/min for up to 24 h. The choice of tirofiban use was left at the discretion of the treating physician but was recommended under conditions with an increased risk of re-occlusion or distal embolization, such as stenting, angioplasty, a high number of passes, or atherosclerotic aetiology. The primary efficacy outcome was the mRS score at 90 days. Safety events according to IVT-treatment status are not reported. However, the authors speculated that the higher mortality and sICH in patients not receiving tirofiban were due to higher frequency of previous anticoagulation, IVT and IAT (even though the last two were included as covariates in the adjusted analyses).

The study by Sun et al.^86^ was a single-centre, retrospective, observational study from China on consecutive 18–80-year-old patients with atherosclerotic BAO who underwent EVT within 24 h of symptom onset between January 2012 and July 2018. Patients with pre-stroke mRS > 1, NIHSS < 10 or >35 (or 0 in the item 1A), significant cerebellar mass effect, bilateral extended brain stem ischaemia, or embolic occlusion were excluded. The treatment groups received either tirofiban (0.3–0.4 mg within 6–8 min IA and 0.15 μg/kg/min IV for 24 h) followed by dual antiplatelet therapy (*n* = 74) or immediate dual antiplatelet therapy (*n* = 31). Tirofiban was used based on the treating physician’s decision in cases with emergency stenting or balloon angioplasty, local new thrombosis or vascular dissection, and severe atherosclerotic lesions with a high risk of re-occlusion. In the tirofiban group, 24.3% received IVT and 20.3% IAT, whereas the rates were 6.5% and 32.3% in the no-tirofiban group. The primary outcomes were 90-day functional independence (mRS 0-2) and favourable functional outcome (mRS 0–3).

Yang et al.^90^ included consecutive adult acute stroke patients with major large artery occlusion undergoing EVT between June 2015 and December 2017 from the Chinese, multicentre, prospective ANGEL registry. The posterior circulation occlusion subgroup (*n* = 158/662) consisted of basilar and dominant vertebral occlusions treated within 24 h of symptom onset, excluding patients with NIHSS < 6 and pre-stroke mRS > 1. Add-on tirofiban (0.25–1 mg IA, followed by 0.1 μg/kg/min IV for 24 h) was considered for patients with emergency stenting or angioplasty, presumed endothelial damage, instant re-occlusion, or severe in situ atherosclerosis with a high risk of early re-occlusion (*n* = 74), whereas the rest did not have add-on tirofiban (*n* = 84). Bridging IVT was used in 23.9% of the tirofiban group and 35.2% of the no-tirofiban group in the whole cohort but the numbers could not be extracted solely for the posterior circulation occlusion subgroup. The primary efficacy endpoints were functional independence (mRS 0–2) and mortality at 90 days, and the primary safety endpoint was sICH at 24-h imaging control.

### Additional information

A study by Pan et al.^87^ was a prospective registry study from two Chinese centres comparing tirofiban (*n* = 64) versus no tirofiban (*n* = 66) as an adjunctive therapy of EVT for patients with vertebral or BA occlusion between October 2016 and July 2021. Tirofiban was administered 0.25–1 mg IA, followed by 0.1–0.15 μg/kg/min IV for 16–24 h at the discretion of the treating physician for patients with severe residual stenosis (⩾50%) after thrombectomy, rescue treatment with stenting or angioplasty, ⩾3 passes, or severe atherosclerosis with a high risk of re-occlusion. IVT was received by 25.0% in the tirofiban and 39.4% in the no-tirofiban group. The outcomes were 90-day mRS score of 0–2, NIHSS at discharge, in-hospital and 90-day mortality, frequency of sICH, and successful recanalisation (TICI ⩾ 2b).

A study by Kellert al.^89^ was a prospective registry study from Germany on consecutive AIS patients treated with EVT in 2006–2011. In the posterior circulation occlusion subgroup, 20 patients received tirofiban IV for at least 12 h according to weight and kidney function (recommended if stenting was performed or endothelial injury was feared) and 14 did not. The IVT rate was 65.0% in the former and 78.5% in the latter group. Outcomes included excellent (mRS 0–1) and good (mRS 0–2) functional outcome at 90 days, sICH rate, mortality, and successful recanalisation (TICI ⩾ 2b).

Zhao et al.^91^ compared patients undergoing EVT who did (*n* = 37 with posterior circulation occlusions) or did not (*n* = 25 with posterior circulation occlusions) receive add-on tirofiban between January 2013 and February 2017 from a Chinese, single-centre, prospective registry. Only patients for whom second-generation stent retrievers were used were included. Tirofiban dosing was 0.25–0.5 mg IA, followed by 0.2–0.25 mg/h for 12–24 h. Typical indications for tirofiban at the interventionists’ discretion were emergency stenting or angioplasty, successful recanalisation by three or more passes, and severe atherosclerosis lesions with high possibility of re-occlusion. In the tirofiban group, 11% received IVT and 24% IAT, whereas the respective numbers were 4% and 19% in the no-tirofiban group. The primary outcome was sICH, and the secondary outcomes included 90-day and long-term functional outcome, mortality, early re-occlusion, and successful recanalisation.

Wu et al.^92^ reported results from a Chinese, non-randomised, single-arm trial with an original plan to give tirofiban to all adult EVT patients within 2 years. However, the trial was stopped after 1 year due to safety concerns (ICH), so during the second year no patients received tirofiban. Thus, the patients treated within the first (*n* = 23/94 with posterior circulation occlusions) and the second year (*n* = 17/124 with posterior circulation occlusions) were compared. The patients with EVT after 24 h from symptom onset or ICH were excluded. Contrary to other studies, tirofiban was administered only as IA boluses with doses depending on the bleeding risk (maximum dose 10 μ/kg). The IVT and IAT rates were not reported for the posterior circulation stroke patients separately but were 16.0% and 4.4% in the whole cohort of tirofiban-treated patients and 30.1% and 4.2% among the patients who did not receive tirofiban. The presence of sICH was the primary outcome complemented by other haemorrhagic outcomes, 90-day functional outcomes, and mortality.

Finally, the study by Ma et al.^93^ was the only one to investigate add-on eptifibatide versus no eptifibatide in patients treated with endovascular approach within 24 h of onset for large-vessel occlusion. The study derived the intervention arm from the Chinese, multicentre, open-label, single-arm EPOCH trial (April 2019 to March 2020) and the control arm from the Chinese, multicentre, prospective ANGEL-ACT registry (November 2017 to March 2019). The former included only patients with mechanical thrombectomy, whereas the latter allowed patients with any EVT including sole IAT. The posterior circulation subgroup comprised 46/162 patients in the propensity score matched cohort, 23 in each treatment arm. Eptifibatide was delivered as 135–180 μg/kg in 5 min IV/IA, followed by 0.75–2 μg/kg/min IV for 24 h. The IVT rate was 25.9% in each treatment arm of the propensity score matched cohort but was not reported for posterior circulation occlusion patients separately. The primary efficacy outcome was 90-day good outcome, defined as mRS score of 0–2, and propensity score matching was used for analyses.

We excluded one retrospective registry study on tirofiban vs. no tirofiban for patients with vertebrobasilar occlusion (86% BAO) treated with endovascular approach within 24 h of onset^94^ due to inconsistent reporting of the results. The authors were contacted several times for clarification, but they did not respond to the request.

**Figure 9.1.**
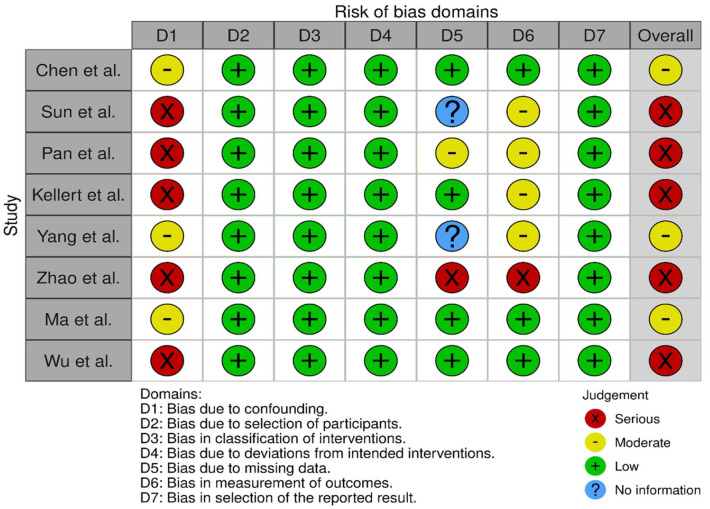
PICO 10 – Risk of bias of the studies included.

The risk of bias is outlined in Figure 9.1. Severity of the risk of confounding bias ranged from moderate to critical. The most common concern appearing in all observational studies was that the add-on antithrombotic agent was chosen based on periprocedural factors that differed systematically between the treatment groups, such as the number of passes or instant re-occlusion, in-situ thrombosis, or residual stenosis requiring emergency angioplasty or stenting.

**Figure 9.2.**
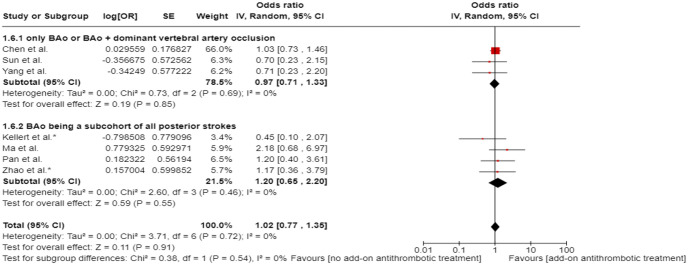
PICO 10 – Metanalysis of observational studies comparing add-on antithrombotic treatment versus no add-on antithrombotic medication stratified by studies with only basilar or dominant vertebral artery occlusion versus studies, where basilar artery occlusion was a subgroup of patients: mRS score of 0–2 at 3 months (pooled OR, random-effects meta-analysis, Cochran’s Q-test for interaction testing). *Unadjusted studies.

We performed a meta-analysis stratified by the proportion of BAO patients within the studies: (a) studies including solely patients with BAO or BAO plus dominant vertebral artery occlusion and (b) studies with a subgroup of BAO patients or uncertain proportion among other posterior circulation strokes (Figures 9.2–9.5).

**Figure 9.3.**
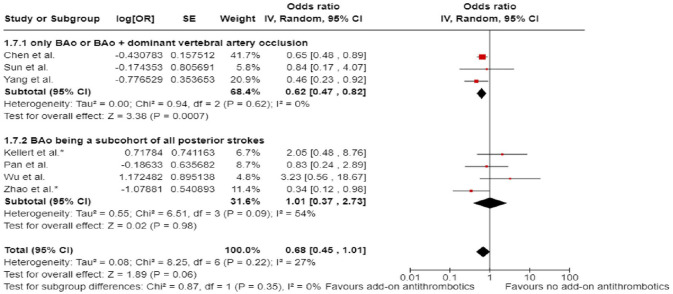
PICO 10 – Metanalysis of observational studies comparing add-on antithrombotic treatment versus no add-on antithrombotic medication stratified by studies with only basilar or dominant vertebral artery occlusion versus studies, where basilar artery occlusion was a subgroup of patients: Mortality (pooled OR, random-effects meta-analysis, Cochran’s Q-test for interaction testing). *Unadjusted studies.

**Figure 9.4.**
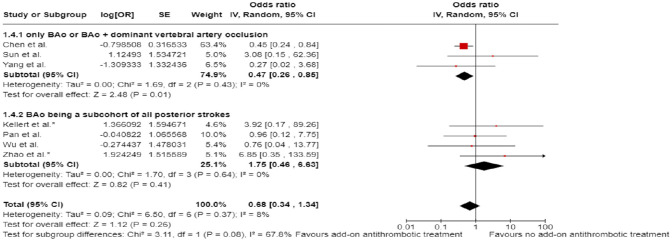
PICO 10 – Metanalysis of observational studies comparing add-on antithrombotic treatment versus no add-on antithrombotic medication stratified by studies with only basilar or dominant vertebral artery occlusion versus studies, where basilar artery occlusion was a subgroup of patients: sICH (pooled OR, random-effects meta-analysis, Cochran’s Q-test for interaction testing). *Unadjusted studies

**Figure 9.5.**
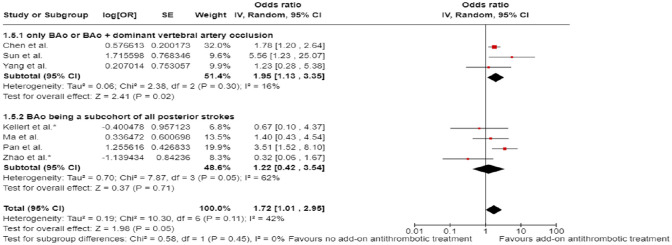
PICO 10 – Metanalysis of observational studies comparing add-on antithrombotic treatment versus no add-on antithrombotic medication stratified by studies with only basilar or dominant vertebral artery occlusion versus studies, where basilar artery occlusion was a subgroup of patients: recanalisation TICI 2B-3 (pooled OR, random-effects meta-analysis, Cochran’s Q-test for interaction testing). *Unadjusted studies.

For both critical outcomes (mortality and sICH) and one important outcome (mTICI 2B/3), the analyses favoured add-on antithrombotic treatment in studies including solely patients with BAO or BAO plus dominant vertebral artery occlusion, whereas no difference was noticed if we included studies, where BAO patients were only a part of posterior circulation strokes. However, it should be noted that the significant findings are mainly based on the study by Chen et al.,^88^ in which no-tirofiban group had a very poor outcome (mortality 52%, sICH 10%). The authors discussed the reliability of their findings and speculated if this was due to higher frequency of previous anticoagulation, IVT, and IAT (even though the last two and cardioembolic aetiology were included as covariates in the adjusted analyses).

Table 6 provides details regarding the assessment of the quality of evidence for PICO 10.

**Table 6.** GRADE evidence profile for PICO 10.

Certainty assessment							No. of patients		Effect		Certainty	Importance
No. of studies	Study design	Risk of bias	Inconsistency	Indirectness	Imprecision	Other considerations	Add-on antithrombotic treatment	No add-on antithrombotic treatment	Relative (95% CI)	Absolute (95% CI)		
mRS 0–2 at 90 days: Observational studies												
7	Observational studies	Serious^a^	Not serious	Not serious	Not serious	None			OR 1.02 (0.77–1.35)	1 fewer per 1000 (from 1 fewer to 1 fewer)	⨁○○○	Important
											Very low	
Mortality at 90 days: Observational studies												
7	Observational studies	Serious^a^	Not serious	Not serious	Not serious	None	222/652 (34.0%)	232/516 (45.0%)	OR 0.68 (0.45–1.01)	92 fewer per 1000 (from 181 fewer to 2 more)	⨁○○○	Critical
											Very low	
Symptomatic intracranial haemorrhage (sICH): Observational studies												
7	Observational studies	Serious^a^	Not serious	Not serious	Not serious	None	31/654 (4.7%)	40/519 (7.7%)	OR 0.68 (0.34–1.34)	23 fewer per 1000 (from 46 fewer to 24 more)	⨁○○○	Critical
											Very low	
mTICI (TICI: Thrombolysis in cerebral ischemia): Observational studies												
7	Observational studies	Serious^a^	Not serious	Not serious	Not serious	None			OR 1.72 (1.01–2.95)	2 fewer per 1000 (from 3 fewer to 1 fewer)	⨁○○○	Important
											Very low	

CI: confidence interval; OR: odds ratio.

a Serious risk of bias due to serious confounding reported in studies implemented for this outcome according to ROBINS-I tool for observational studies.


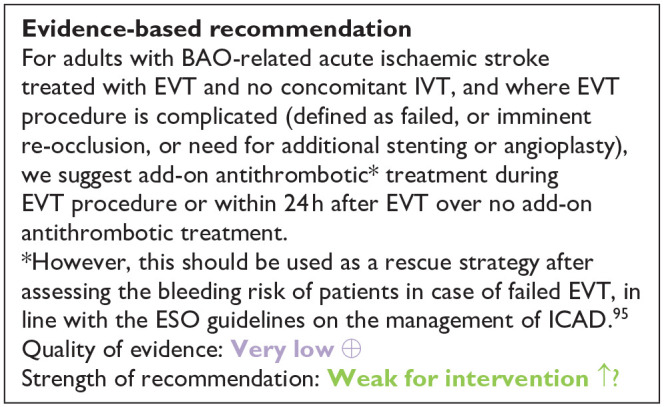


## Discussion

This guideline has been developed following the GRADE methodology and it aims to assist physicians in decision-making in the acute management of BAO. All recommendations and Expert Consensus Statements are summarised in Table 7. Whenever possible, we based our recommendations on RCTs or meta-analyses of RCTs. However, we found that randomised data were mostly scarce or lacking. This was expected given the catastrophic prognosis of BAO, due to which randomised trials of reperfusion therapies compared to conventional treatment (comprising antiplatelets or anticoagulation) may not be considered ethical. Hence, we also used data from NRSIs, which are more prone to selection bias and confounding, however, we followed the Cochrane recommendations for combining data from RCTs and NRSIs.

**Table 7.** Synoptic table of all recommendations and Expert Consensus Statements.

Recommendation	Expert consensus statement (10 voting members)
PICO 1 for adults with BAO-related acute ischaemic stroke presenting within 24 h from the time last known well, does intravenous thrombolysis (IVT) alone compared to no IVT improve outcomes?	
For adults with BAO-related acute ischaemic stroke presenting within 24 h from the time last known well, there are insufficient data to make an evidence-based recommendation on the use of IVT. Please see the Expert Consensus Statement below. Quality of evidence: - Strength of recommendation: -	1. For adults with BAO-related acute ischaemic stroke presenting within 4.5 h from the time last known well without contraindications for IVT and without extensive ischemic changes in the posterior circulation*, 10/10 MWG members suggest intravenous thrombolysis rather than no intravenous thrombolysis (please also see PICO 5 and 7). 2. For adults with BAO-related acute ischaemic stroke presenting between 4.5 and 12 h from the time last known well without contraindications for IVT (apart from the time window) and without extensive ischemic changes in the posterior circulation*, 8/10 MWG members suggest intravenous thrombolysis rather than no intravenous thrombolysis (please also see PICO 5 and 7). 3. For adults with BAO-related acute ischaemic stroke presenting between 12 and 24 h from the time last known well without contraindications for IVT (apart from the time window) and without extensive ischemic changes in the posterior circulation*, 8/10 MWG members suggest intravenous thrombolysis rather than no intravenous thrombolysis (please also see PICO 5 and 7). *extensive bilateral and/or brainstem ischemic changes
PICO 2 For adults with BAO-related acute ischaemic stroke within 6 h of symptoms onset, does endovascular treatment (EVT) plus best medical treatment (BMT) compared with BMT alone improve outcomes?	
For adults with BAO-related acute ischaemic stroke presenting within 6 h from the time last seen well, we suggest EVT plus BMT over BMT alone*. However, there are caveats, and this recommendation does not apply to all patients as detailed below. The recommendation considers only patients with NIHSS ⩾ 10 (please see also PICO 4). *The effect of treatment depends on use of IVT in BMT group, with greater benefit of EVT seen in those trials with lesser use of IVT. Actually, much of this evidence comes from Asian trials with high prevalence of ICAD, and in which BMT often comprises conventional therapy only (antiaggregatory and anticoagulation). For imaging criteria, please refer to PICO 5). Quality of evidence: **Very low** ⊕ Strength of recommendation: **Weak for intervention** ↑?	
PICO 3 for adults with BAO-related acute ischaemic stroke 6–24 h from the time last known well, does EVT plus BMT compared with BMT alone improve outcomes?	
For adults with BAO-related acute ischaemic stroke presenting within 6–24 h from the time last known well, we suggest EVT plus BMT over BMT alone.* However, there are caveats, and this recommendation does not apply to all patients as detailed below. The recommendation considers only patients with NIHSS ⩾ 10 (please see also PICO 4). * Much of this evidence comes from Asian trials with high prevalence of ICAD, and in which BMT often comprises conventional therapy only (antiaggregatory and anticoagulation). For imaging criteria, please refer to PICO 5. Quality of evidence: **Very low** ⊕ Strength of recommendation: **Weak for intervention** ↑?	
PICO 4 for adults with BAO-related acute ischemic stroke, does selection of reperfusion treatment (IVT or EVT) based on specific presentation (e.g. high NIHSS cutoff, coma on admission, proximal location of basilar artery occlusion) compared with other presentation features (e.g. low NIHSS cutoff, no coma on admission, distal location of basilar artery occlusion) modify the outcome?	
For adults with BAO-related acute ischaemic stroke, there is a differential treatment effect (a significant interaction) of reperfusion therapy according to specific presentation. The treatment effect is different for patients with high compared to low NIHSS scores and for proximal or middle locations of basilar artery occlusions compared to distal locations. (See also PICO 2 and 3 for caveats in general recommendations). For patients presenting with severe symptoms (NIHSS ⩾ 10), we suggest BMT + EVT over BMT only*. *The effect is stronger for proximal and middle location of the occlusion. Quality of evidence: **Very low** ⊕ Strength of recommendation: **Weak for intervention** ↑? For patients presenting with mild-to-moderate symptoms (NIHSS < 10), we could not find evidence to recommend EVT over BMT for efficacy, but BMT appeared safer than EVT. We suggest BMT only over EVT+BMT in this group*. *These data come from a randomised trial with low prevalence of ICAD, and in which BMT very often comprised intravenous thrombolysis. These findings are also supported by non-randomised data. Quality of evidence: **Very low** ⊕ Strength of recommendation: **Weak for intervention** ↑?	
PICO 5 For adults with BAO-related acute ischaemic stroke, does selection of reperfusion therapy (IVT and/or EVT) candidates based on a particular pc-ASPECTS compared with no specific threshold improve identification of patients with a therapy effect on outcomes?	
For adults with BAO-related acute ischaemic stroke without extensive ischaemic changes at baseline (pc-ASPECTS 7–10), we suggest reperfusion therapy over no reperfusion therapy according to the certainty of evidence and strength of recommendation in PICOs 1, 2, 3, 4, and 7. For adults with BAO-related acute ischaemic stroke with pc-ASPECTS 0-6, there are insufficient data to make an evidence-based recommendation on the use of reperfusion therapy. (See the Expert Consensus Statement below). Quality of evidence: - Strength of recommendation: -	For adults with BAO-related acute ischaemic stroke with ischaemic changes at baseline being more extensive than those included in randomised controlled clinical trials (i.e. pc-ASPECTS 0-6), 10/10 MWG members suggest considering other prognostic variables (such as pre-stroke handicap, age, frailty) before offering reperfusion therapy. However, for patients with very extensive bilateral and/or brainstem ischemic lesions, 7/10 MWG members suggest no reperfusion therapy
PICO 6 for adults with BAO-related acute ischaemic stroke, does selection of reperfusion therapy (EVT or IVT) candidates based on advanced imaging criteria (perfusion, core, or collateral imaging) compared with no advanced imaging improve identification of patients with a therapy effect on outcomes?	
For adults with BAO-related acute ischaemic stroke, there are insufficient data to make an evidence-based recommendation on the selection of reperfusion therapy based on evaluation of advanced imaging (perfusion, core, or collateral imaging). Please see the Expert Consensus Statement below. Quality of evidence: - Strength of recommendation: -	For adults with BAO-related acute ischaemic stroke (and in the absence of extensive ischaemic changes in the posterior circulation*), 10/10 MWG members suggest reperfusion therapy (EVT or IVT) rather than no reperfusion therapy, irrespective of any collateral score points. *extensive bilateral and/or brainstem ischemic changes
PICO 7 for adults with BAO-related acute ischaemic stroke without contraindication for IVT, does direct EVT compared to EVT plus IVT improve outcomes?	
For adults with BAO-related acute ischaemic stroke, we suggest combined IVT and EVT treatment over direct EVT in case IVT is not contraindicated. Quality of evidence: **Low** ⊕⊕ Strength of recommendation: **Weak for intervention** ↑?	
PICO 8 for adults with BAO-related acute ischaemic stroke, does mechanical thrombectomy using direct aspiration as the first-line strategy compared with a stent retriever as the first-line strategy improve outcomes?	
For adults with BAO-related acute ischaemic stroke, we suggest EVT using direct aspiration over stent retriever as the first-line strategy. Quality of evidence: **Very low** ⊕ Strength of recommendation: **Weak for intervention** ↑?	
PICO 9 for adults with BAO-related acute ischaemic stroke and with suspected intracranial atherosclerotic disease (ICAD) and BA stenosis, does PTA and/or stenting of the basilar artery plus EVT compared with EVT alone improve outcomes?	
For adults with BAO-related acute ischaemic stroke and with a suspected ICAD and BA stenosis, there is insufficient evidence to make an evidence-based recommendation on the use of PTA and/or stenting in addition to EVT. Please see the Expert Consensus Statement below. Quality of evidence: - Strength of recommendation: -	For adults with BAO-related acute ischaemic stroke and with suspected ICAD and severe underlying BA stenosis, 10/10 MWG members suggest rescue PTA and/or stenting after failed endovascular procedure (please also see PICO 10).
PICO 10 For adults with BAO-related acute ischaemic stroke subjected to reperfusion therapy (EVT or IVT), does add-on antithrombotic treatment during EVT or within 24 h after IVT or EVT compared with no add-on antithrombotic treatment improve outcomes?	
For adults with BAO-related acute ischaemic stroke treated with EVT and no concomitant IVT, and where EVT procedure is complicated (defined as failed, or imminent re-occlusion, or need for additional stenting or angioplasty), we suggest add-on antithrombotic* treatment during EVT procedure or within 24 h after EVT over no add-on antithrombotic treatment. *However, this should be used as a rescue strategy after assessing the bleeding risk of patients in case of failed EVT, in line with the ESO guidelines on the management of ICAD.^95^ Quality of evidence: **Very low** ⊕ Strength of recommendation: **Weak for intervention** ↑?	

Cochrane methodology, GRADE, is the cornerstone of ESO guidelines. The rigorous approach of this methodology can explain the very low quality of evidence for EVT in PICO 2 and 3. The robustness of this system is underscored by the fact that the same evaluation was performed in other available meta-analyses of the same RCTs, including investigators from China.^96–98^ According to a recent meta-analysis of RCTs, the associations reported in the Asian trials were not robust, as indicated by a low fragility index for every outcome and heterogeneity.^98^ We also want to point out some general observations. First, the few existing RCTs were mostly (three out of four trials) performed in Asian populations with a high prevalence of ICAD compared to other populations. In these trials, EVT was compared to BMT, which included IVT only in every fourth to every third patient. According to the investigators, the latter was linked to the fact that some patients had to initially pay for the IVT. Furthermore, there might also be some differences in the system of care in patients who underwent EVT compared to those in the BMT arm. Two of these trials were positive,^8,9^ and one was neutral,^6^ with a very high crossover rate. In contrast, the BASICS trial^7^ randomised patients in 23 centres, of which 20 were in Europe and 3 in Brazil. In this trial, 80% of patients in the BMT arm received IVT, and there was no difference in functional outcome between the arms. Second, no superiority of EVT was observed in the subgroup analyses of ATTENTION and BAOCHE RCTs, when BMT included solely IVT-treated patients. Third, the direction of the treatment effect in the forest plots of the RCTs and NRSIs were largely determined by the proportion of IVT in the BMT arms, which was further confirmed by interaction analyses. Finally, the ATTENTION and BAOCHE trials used more restrictive inclusion criteria and selected patients with a more favourable profile toward EVT-associated efficacy. This includes a prolonged time window, younger patients with minimum pre-stroke disability, and no significant ischaemic changes on baseline imaging. Consequently, generalizing the findings to other patient populations may be questionable.

Regarding another set of interaction analyses investigating the potential treatment-modifying effect on NIHSS scores, we would like to point out that the interaction analyses of this variable were typically reported in 2 or 3 categories with various cutoffs values between different studies. We observed a significant treatment-modifying effect stratified by a baseline NIHSS score of 10, favouring BMT for patients with NIHSS < 10. This is in line with a recently published meta-analysis of two RCTs.^30^ If we look at the data from the Asian RCTs, we notice that the majority of the recruited patients had extremely severe clinical symptoms on admission. In the BEST trial, the median NIHSS in the EVT arm was 32, which gives us a better understanding of the population of patients to whom the results of these trials apply. Indeed, the ATTENTION investigators stated that their results are not generalizable for patients with an NIHSS of less than 10. The effect of EVT was more visible in proximal and middle locations but less in distal occlusions.

The next block of PICO questions addressed the possible treatment-modifying effect of recanalisation therapy stratified by early ischaemic signs, collateral flow, core, and perfusion imaging. Mostly consensus-level recommendations were given, but future research may evaluate treatment-modifying effect of novel collateral scores^99^ or scores combining the collateral status and early ischaemic changes.^100^

Similar to anterior circulation strokes,^101^ we also observed better outcomes of combined IVT + EVT over direct EVT approach. In technical terms, we suggest direct aspiration over stent-retriever as the first-line strategy. New trials are needed to find evidence whether EVT under general anaesthesia leads to better outcome than with no general anaesthesia, however, very recent data from the post-hoc analysis of the BASICS RCT suggest that early intubation was linked to unfavourable outcomes.^102^ In a consensus statement, the MWG suggests rescue PTA and/or stenting after a failed EVT procedure. The ANGEL-REBOOT RCT could bring some more light into this issue. Finally, there are no evidence-based data on the add-on antithrombotic treatment during or after recanalisation therapies. Such evidence should be derived from RCTs. In situations where inclusion in a dedicated RCT is not possible, we suggest (with a very low level of evidence) that in the case of complicated EVT (defined as failed, or imminent re-occlusion, or need for additional stenting or angioplasty), add-on antithrombotic treatment may be used. However, this should be employed as a rescue strategy after assessing the bleeding risk of patients in the event of unsuccessful EVT, in line with the ESO guidelines on the management of ICAD.^95^

In conclusion, this ESO guideline aims to address the primary clinical questions on the acute management of patients with BAO, which is associated with one of the worst natural outcomes among stroke patients. Unlike other guidelines, we do not anticipate the availability of new randomised data specifically for this stroke subtype in the near future. However, we might see a comparison between alteplase and tenecteplase, and there is potential for individual patient data pooled analysis from some of the RCTs and/or registries, which could provide new insights in the future.

## Plain language summary

The basilar artery supplies blood to the back of the brain and brainstem, including critical areas involved in the regulation of breathing, consciousness, swallowing, vision, and mobility. Individuals who suffer an ischaemic stroke due to a blood clot in the basilar artery, have a very high risk of death or permanent disability if the clot cannot be dissolved or removed rapidly. The two treatment strategies aimed at acute clot busting or removal are administration of clot-dissolving drugs into a vein (intravenous thrombolysis) and mechanical removal of the clot with a catheter placed into an artery (mechanical thrombectomy). However, these treatments also carry risks, such as bleeding in the brain, and they can be ineffective if given too late. This guideline provides recommendations for the acute treatment of stroke caused by basilar artery occlusion using clot-busting or removal therapies.

The key recommendations/suggestions of the guideline include the following:

1. Treat patients with basilar artery occlusion with intravenous thrombolysis within 24 h of symptom onset if there are no contraindications, such as extensive, already permanent ischaemic damage to the brain. Thrombolysis should be used regardless of the severity of stroke symptoms.
2. Treat patients with basilar artery occlusion and moderate-to-severe stroke symptoms with mechanical thrombectomy within 24 h of symptom onset if there is not extensive, already permanent ischaemic damage to the brain. Patients with mild stroke symptoms may experience harm from thrombectomy.
3. Use intravenous thrombolysis in addition to mechanical thrombectomy if there are no contraindications.
4. Choose direct suction of the clot with an aspiration catheter as the first-line approach in mechanical thrombectomy, instead of a stent retriever.

Some of the recommendations and suggestions about mechanical thrombectomy for patients with symptoms due to basilar artery clot were supported by very low-quality evidence, whereas the rest were based on expert opinions.

## Supplemental Material

sj-docx-1-eso-10.1177_23969873241257223 – Supplemental material for European stroke organisation and European society for minimally invasive neurological therapy guideline on acute management of basilar artery occlusion Supplemental material, sj-docx-1-eso-10.1177_23969873241257223 for European stroke organisation and European society for minimally invasive neurological therapy guideline on acute management of basilar artery occlusion by Daniel Strbian, Georgios Tsivgoulis, Johanna Ospel, Silja Räty, Petra Cimflova, Georgios Georgiopoulos, Teresa Ullberg, Caroline Arquizan, Jan Gralla, Kamil Zeleňák, Salman Hussain, Jens Fiehler, Patrik Michel, Guillaume Turc and Wim Van Zwam in European Stroke Journal
