# Supplementary material for: European Stroke Organisation and European Society for Minimally Invasive Neurological Therapy guideline on acute management of basilar artery occlusion
Source: Eur Stroke J. 2024 Jul 22;9(4):835–84. doi: 10.1177/23969873241257223 (PMC11569583; doi:10.1177/23969873241257223)
Supplement: sj-docx-1-eso-10.1177_23969873241257223 – Supplemental material for European stroke organisation and European society for minimally invasive neurological therapy guideline on acute management of basilar artery occlusion [file sj-docx-1-eso-10.1177_23969873241257223.docx]

Supplemental Table 1. Conflicts of interest of module working group members

| **Author** | **Discipline and affiliation** | **Intellectual and financial disclosures** |
| --- | --- | --- |
| Daniel Strbian | Department of Neurology  HUS Neurocenter, Helsinki University Hospital and University of Helsinki, Helsinki, Finland | Intellectual disclosures:  -European Stroke Organization (ESO): Executive committee  -Member of the ESO Guideline board  -National Coordinator of SITS registry  -Assistant Editor “Stroke” Journal  -Co-author of ESO Guideline on IVT for AIS (PMID: 33817340)  -Co-author of ESO Guideline on IVT before MT in AIS pts with LVO (PMID: 35342811)  -Steering committee of RCTs: DISTAL, PROOF, SWIFT-DIRECT, ELAN, SWITCH, Milvexian SSP, Librexia, TECNO, ICARUS  Financial disclosures:  -Advisory Board: Astra-Zeneca, Alexion, CSL Behring, Shionogi, BMS, Janssen  -Unrestricted Research or Educational Grants: Boehringer-Ingelheim |
| Caroline Arquizan | Department of Neurology, Stroke Unit, Montpellier University Hospital | Steering commitee of RCT In EXTREMIS (MOSTE and LASTE) which was funded by Montpellier University Hospital through an unrestricted grant from an industry consortium (MEDTRONIC, STRYKER, BALT EXTRUSION, MICROVENTION, CERENOVUS)  Intellectual disclosure : General Secretary of the Societe Française NeuroVascualire  Personal honoraria from speaker honoraria from MEDTRONIC, AMGEN |
| Petra Cimflova | MD, PhD, Radiologist  Department of Radiology, University of Calgary, Calgary, Canada  Department of Medical Imaging, St. Anne’s University Hospital Brno and Faculty of Medicine MU Brno, Brno, Czech Republic  Klinik für Neuroradiologie, Universitaetsklinikum Freiburg, Breisacher Strasse 64, 79106 Freiburg, Germany | None |
| Jens Fiehler | Neuroradiology, UMC Hamburg-Eppendorf | Consultant for Acandis, Cerenovus, Medtronic, Microvention, Penumbra, Phenox, Roche, Stryker. Stocks of Tegus, Eppdata and Vastrax. |
| Georgios Georgiopoulos | MD, PhD, Cardiologist  Department of Physiology, School of Medicine, University of Patras, Grece and School of Biomedical Engineering and Imaging Sciences, King’s College London, UK | None |
| Jan Gralla | MD, MSC  Department of Diagnostic and Interventional Neuroradiology, Inselspital  University of Bern, Switzerland | Global PI of STAR (NCT01327989) and Swift Direct (NCT03192332) (Medtronic), Consultancy  Consultancy for Johnson & Johnson/Cerenovus |
| Patrik Michel | Neurology Service, Department of Clinical Neurosciences, Lausanne University Hospital and University of Lausanne, Lausanne, Switzerland | No funding related to this project.  Unrelated: Research support to my institution from the Swiss National Science Foundation, the Swiss Heart Foundation, and Faculty of Biology and Medicine of the Lausanne University  Intellectual: member of the Steering committee, and local PI of the BASICS trial |
| Johanna Ospel | Neuroradiology, Department of Diagnostic Imaging, Foothills Medical Centre, University of Calgary, Alberta, Canada | Consultant to Nicolab (unrelated to the guidelines) |
| Silja Räty | Neurology, Department of Neurology, Helsinki University Hospital, Finland | None |
| Georgios Tsivgoulis | Second Department of Neurology, "Attikon" University Hospital, School of Medicine, National and Kapodistrian University of Athens, Athens, Greece | Intellectual disclosures:  -European Stroke Organization (ESO): Vice President  -Hellenic Neurological Society: President  -Hellenic Society of Cerebrovascular Diseases: General Secretary  -Member of the ESO Guideline board  -Chair of ESO Industry Roundtable  -National Coordinator of SITS, ANGELS, RES-Q registries  -Member of SITS Scientific Committee  -Section Editor “Stroke” Journal  -Associate Editor: Therapeutic Advances in Neurological Disorders, Journal of Neuroimaging  -Co-author of ESO Guideline on IVT for AIS (PMID: 33817340)  -Co-author of ESO Guideline on IVT before MT in AIS pts with LVO (PMID: 35342811)  -Co-author of ESO Guideline on Tenecteplase for patients with acute ischaemic stroke (doi: 10.1177/23969873221150022)  -Adjudication committee of BI 1123-0040 RCT (Phase 3 trial evaluating safety and efficacy of TNK vs. TPA in AIS)  -Steering committee of DISTAL RCT (Phase 3 trial evaluating the safety and efficacy of EVT in DMVOs)  Financial disclosures:  -Participation in Advisory Meetings & Satellite Symposia: Novartis, Sanofi, Biogen, Genesis Pharma, Teva, Shire, Merck, Bayer, Daichii-Sankyo, Allergan, Specifar, Actavis, Boehringer-Ingelheim, Medtronic, CSL Behring, Abbott, Takeda, Abbvie, Ipsen, ITF, Shionogi, Novasignal, BMS, Roche, Medison, Astra  -Unrestricted Research or Educational Grants: Novartis, Genesis Pharma, Teva, Shire, Merck, Abbott, Allergan, Boehringer-Ingelheim, Medtronic, Amicus, Abbvie, Ipsen, Bayer, Roche, Novalis |
| Guillaume Turc | GHU Paris Psychiatrie et Neurosciences, 1 rue Cabanis, 75014 Paris, France | Lecture fees: Guerbet France |
| Salman Hussain | European Stroke Organisation, Basel, Switzerland | None |
| Teresa Ullberg | M.D, PhD, Neurology, Department of Clinical Sciences Lund, Lund University, Skane University Hospital, Lund and Malmö, Sweden | TU received personal honoraria from an Expert assignment for Astra Zeneca, and speaker honoraria from Siemens Healthineers. |
| Kamil Zeleňák | Clinic of Radiology, Jessenius Faculty of Medicine, Comenius University, Kollárova 2, 03659 Martin, Slovakia. | Steering committee of the TENSION study - TENSION has received funding from the European Union’s Horizon 2020 research and innovation programme under grant agreement No 754640. |
| Wim van Zwam | Department of Radiology and Nuclear Medicine , Maastricht University Medical Center  Maastricht, The Netherlands | Co P.I. of MrClean and MrClean-Late studies, which received funding from the Dutch Heart Foundation, Health Holland, Dutch Brain Council, Medtronic, Penumbra, Stryker and Cerenovus.  Speaker fees from: Stryker, Cerenovus, NicoLab and Philips, all paid to institution.  DSMB chair: WeTrust, InExtremis, ANAIS |

**Supplemental Table 2. List and rating (mean score) of the selected outcomes for each PICO question.**

| **Outcome / PICO** | **1** | **2** | **3** | **4** | **5** | **6** | **7** | **8** | **9** | **10** |
| --- | --- | --- | --- | --- | --- | --- | --- | --- | --- | --- |
| mRS 0-3 at 3 months | 7.7 | 7.8 | 7.8 | 7.4 | 7.2 | 6.9 | 7.5 | 7.0 | 7.4 | 7.4 |
| mRS 0-2 at 3 months | 8.0 | 7.5 | 7.5 | 7.1 | 7.0 | 6.7 | 7.0 | 6.7 | 6.9 | 6.9 |
| shift mRS at 3 months | 8.4 | 8.4 | 8.4 | 8.0 | 7.9 | 7.6 | 8.1 | 7.7 | 7.9 | 7.9 |
| mortality at 3 months | 6.9 | 7.2 | 7.2 | 7.0 | 7.0 | 6.7 | 6.9 | 6.4 | 6.9 | 7.1 |
| sICH | 6.4 | 6.2 | 6.2 | 5.6 | 5.7 | 5.6 | 6.5 | 6.1 | 6.4 | 7.1 |
| mTICI 2B/3 | 4.8 | 5.2 | 5.2 | 4.7 | 4.9 | 4.8 | 5.3 | 6.2 | 6.8 | 6.1 |

**Supplemental Table 3. Literature search**

***Ovid MEDLINE and Embase(R) ALL <1946 to January 13, 2023>***

| # | search string |
| --- | --- |
| 1 | exp Basilar Artery/ |
| 2 | basilar.ti,ab,kw. |
| 3 | exp Arterial Occlusive Diseases/ |
| 4 | 'basilar artery occlusion'.mp. |
| 5 | 'basilar artery obstruction'.mp. |
| 6 | 'acute basilar artery occlusion'.mp. |
| 7 | artery occlusion.mp. |
| 8 | blood vessel occlusion.mp. |
| 9 | BAO.mp. |
| 10 | Occlusion.mp. |
| 11 | Occlusions.mp. |
| 12 | 'basilar artery occlusions'.mp. |
| 13 | Vertebrobasilar Insufficiency/ |
| 14 | vertebrobasilar occlusion.mp. |
| 15 | vertebrobasilar.mp. |
| 16 | vertebrobasilar circulation.mp. |
| 17 | posterior circulation.mp. |
| 18 | 'posterior cerebral'.mp. |
| 19 | vertebral.mp. |
| 20 | "pc-ASPECTS ".mp. |
| 21 | "Posterior Circulation ASPECTS".mp. |
| 22 | 'Basilar Artery International Cooperation Study'.mp. |
| 23 | or/1-22 |
| 24 | cerebrovascular accident.mp. |
| 25 | cerebrovascular disorders/ or basal ganglia cerebrovascular disease/ or exp brain ischemia/ or carotid artery diseases/ or carotid artery thrombosis/ or intracranial arterial diseases/ or cerebral arterial diseases/ or exp "intracranial embolism and thrombosis"/ or exp stroke/ |
| 26 | (isch?emi$ adj6 (stroke$ or apoplex$ or cerebral vasc$ or cerebrovasc$ or cva)).tw. |
| 27 | ((brain or cerebr$ or cerebell$ or vertebrobasil$ or hemispher$ or intracran$ or intracerebral or infratentorial or supratentorial or middle cerebr$ or mca$ or anterior circulation) adj5 (isch?emi$ or infarct$ or thrombo$ or emboli$ or occlus$ or hypoxi$)).tw. |
| 28 | ((brain$ or cerebr$ or cerebell$ or intracerebral or intracran$ or parenchymal or intraparenchymal or intraventricular or infratentorial or supratentorial or basal gangli$ or putaminal or putamen or posterior fossa or hemispher$ or subarachnoid) adj5 (h?emorrhag$ or h?ematoma$ or bleed$)).tw. |
| 29 | or/24-28 |
| 30 | 23 and 29 |
| 31 | radiography, interventional/ or radiology, interventional/ |
| 32 | catheterization/ or angioplasty/ or angioplasty, balloon/ or angioplasty, balloon, laser-assisted/ or angioplasty, laser/ or atherectomy/ or catheter ablation/ |
| 33 | Stents/ |
| 34 | mechanical thrombolysis/ or thrombectomy/ or embolectomy/ |
| 35 | endovascular thrombectomy.mp. |
| 36 | endovascular therapy.mp. |
| 37 | endovascular treatment.mp. |
| 38 | 'NIHSS score'.mp. |
| 39 | blood vessel prosthesis/ or blood vessel prosthesis implantation/ |
| 40 | cerebral revascularization/ or reperfusion/ or dilatation/ |
| 41 | (interventional adj3 (radiolog$ or radiograph$ or neuroradiolog$)).tw. |
| 42 | (angioplast$ or stent$).tw. |
| 43 | (thrombectomy or embolectomy or atherect$).tw. |
| 44 | (thromboaspiration or arterial recanali?ation).tw. |
| 45 | ((mechanical or radiolog$ or pharmacomechanical or laser or endovascular or neurovascular) adj5 (thrombolys$ or reperfusion or fragmentation or aspiration or recanali?ation or clot lys$)).tw. |
| 46 | ((clot or thrombus or thrombi or embol$) adj5 (aspirat$ or remov$ or retriev$ or fragment$ or retract$ or extract$ or obliterat$ or dispers$ or disrupt$ or disintegrate$)).tw. |
| 47 | ((retrieval or extraction) adj5 device$).tw. |
| 48 | endoluminal repair$.tw. |
| 49 | ((merci or concentric) adj retriever).tw. |
| 50 | (endovascular snare$ or neuronet or microsnare or X-ciser or angiojet).tw. |
| 51 | thrombolytic therapy/ |
| 52 | fibrinolytic agents/ or fibrinolysin/ or plasminogen/ or tissue plasminogen activator/ or exp plasminogen activators/ or urokinase-type plasminogen activator/ or exp streptokinase/ |
| 53 | fibrinolysis/ |
| 54 | (thromboly$ or fibrinoly$ or recanalis$ or recanaliz$).tw. |
| 55 | ((clot$ or thrombus) adj5 (lyse or lysis or dissolve$ or dissolution or bust$)).tw. |
| 56 | (tPA or t-PA or rtPA or rt-PA or plasminogen or plasmin or alteplase or actilyse).tw. |
| 57 | (tPA or t-PA or rtPA or rt-PA or plasminogen or plasmin or alteplase or actilyse).nm. |
| 58 | (anistreplase or streptodornase or streptokinase or urokinase or pro?urokinase or rpro?uk or lumbrokinase or duteplase or lanoteplase or pamiteplase or reteplase or saruplase or staphylokinase or streptase or tenecteplase or desmoteplase or amediplase or monteplase or nasaruplase or silteplase).tw. |
| 59 | (anistreplase or streptodornase or streptokinase or urokinase or pro?urokinase or rpro?uk or lumbrokinase or duteplase or lanoteplase or pamiteplase or reteplase or saruplase or staphylokinase or streptase or tenecteplase or desmoteplase or amediplase or monteplase or nasaruplase or silteplase).nm. |
| 60 | or/31-59 |
| 61 | Epidemiologic Studies/ |
| 62 | exp Case Control Studies/ |
| 63 | exp Cohort Studies/ |
| 64 | (epidemiologic adj (study or studies)).ab,ti. |
| 65 | case control.ab,ti. |
| 66 | (cohort adj (study or studies)).ab,ti. |
| 67 | cohort analy$.ab,ti. |
| 68 | (follow up adj (study or studies)).ab,ti. |
| 69 | longitudinal.ab,ti. |
| 70 | retrospective$.ab,ti. |
| 71 | prospective$.ab,ti. |
| 72 | (observ$ adj3 (study or studies)).ab,ti. |
| 73 | registry study.mp. |
| 74 | randomised controlled trial.pt. |
| 75 | controlled clinical trial.pt. |
| 76 | randomised.ab. |
| 77 | placebo.ab. |
| 78 | clinical trials as topic.sh. |
| 79 | randomly.ab. |
| 80 | trial.ti. |
| 81 | or/61-80 |
| 82 | 30 and 60 and 81 |
| 83 | exp animals/ not humans.sh. |
| 84 | 82 not 83 |

**Final hits: 11766**
